# Evaluation of Site-Specific Radioiodination of Anti-EGFR-Targeted DARPin E01 Using (4-Hydroxyphenyl)ethyl Maleimide

**DOI:** 10.3390/ijms27167438

**Published:** 2026-08-20

**Authors:** Mariia Larkina, Gleb Yanovich, Lutfi A. Hasnowo, Ruslan Varvashenya, Daria Eskova, Ivan Sharychev, Anastasia Prach, Evgenii Plotnikov, Roman Zelchan, Alexey Schulga, Elena Konovalova, Rustam H. Ziganshin, Mikhail Belousov, Vladimir Tolmachev, Sergey M. Deyev

**Affiliations:** 1 Research Centrum for Oncotheranostics, Research School of Chemistry and Applied Biomedical Sciences, Tomsk Polytechnic University, 634050 Tomsk, Russia; marialarkina@mail.ru (M.L.); sonne_gleb@mail.ru (G.Y.); lutf006@brin.go.id (L.A.H.); mr.varvashenya@mail.ru (R.V.); eskova@tpu.ru (D.E.); sharychev@tpu.ru (I.S.); nastya.prach@mail.ru (A.P.); plotnikovev@tpu.ru (E.P.); r.zelchan@yandex.ru (R.Z.); schulga@gmail.com (A.S.); elena.ko.mail@gmail.com (E.K.); biomem@mail.ru (S.M.D.); 2 Department of Pharmaceutical Analysis, Siberian State Medical University, 634050 Tomsk, Russia; 3 Polytechnic Institute of Nuclear Technology, National Research and Innovation Agency of Indonesia, Yogyakarta 55281, Indonesia; 4 Department of Nuclear Medicine, Cancer Research Institute, Tomsk National Research Medical Center, Russian Academy of Sciences, 634009 Tomsk, Russia; 5 Molecular Immunology Laboratory, Shemyakin & Ovchinnikov Institute of Bioorganic Chemistry, Russian Academy of Sciences, 117997 Moscow, Russia; 6 Collective Use Center “Bioorganic”, Shemyakin & Ovchinnikov Institute of Bioorganic Chemistry, Russian Academy of Sciences, 117997 Moscow, Russia; ziganshin@mail.ru; 7 Department of Immunology, Genetics and Pathology, Uppsala University, 75185 Uppsala, Sweden

**Keywords:** EGFR, DARPin E01, radioiodination, HPEM, SPECT, scaffold protein, biodistribution, site-specific labelling

## Abstract

Non-invasive radionuclide molecular imaging of epidermal growth factor receptor (EGFR) expression can guide patient stratification for EGFR-targeted therapies. The designed ankyrin repeat protein (DARPin) E01, which binds EGFR ectodomain III with sub-nanomolar affinity, is a promising scaffold for single-photon emission computed tomography (SPECT) imaging probes. In the present study, we compared site-specific radioiodination of DARPin E01 using the bifunctional prosthetic group (4-hydroxyphenyl)ethyl maleimide (HPEM) with site-unspecific radioiodination via [^123^I]I-para-iodobenzoate (PIB). [^123^I]I-HPEM was conjugated to the C-terminus of DARPin E01 via Glu-Glu-Glu-Cys ([^123^I]I-E01-E_3_C-HPEM) or Gly-Gly-Gly-Cys ([^123^I]I-E01-G_3_C-HPEM) linkers. Radiolabelling yields were 6 ± 2% and 13 ± 5%, respectively. Size-exclusion purification provided radiochemical purity > 98%. Both HPEM conjugates retained nanomolar EGFR-binding affinity (K_D_: 3.2 ± 0.6 and 4.8 ± 0.9 nM) and demonstrated EGFR-specific tumour accumulation in A-431 xenografts. Cellular processing was characterised by rapid binding, slow internalisation, and non-residualising behaviour of all variants. Kidney uptake was lower for the site-specifically labelled variants. However, site-specific labelling evidently elevated hepatobiliary excretion and uptake in Na/I-symporter-expressing organs compared to [^123^I]I-(HE)_3_-E01-PIB, while linker composition (E_3_C vs. G_3_C) did not significantly alter biodistribution. Site-unspecific radioiodination with [^123^I]I-PIB remains the preferred approach for clinical SPECT imaging of EGFR expression with DARPin E01.

## 1. Introduction

Targeted therapeutic intervention with highly selective drugs is a powerful approach to treating disseminated malignancies [1]. Epidermal growth factor receptor (EGFR), a transmembrane tyrosine kinase belonging to the ErbB (HER) receptor family, regulates key cellular processes—including proliferation, survival, motility, and oncogenic transformation—through downstream signalling cascades activated upon ligand binding. EGFR is aberrantly overexpressed or activated in a broad spectrum of epithelial malignancies [1,2]. Elevated EGFR expression is associated with unfavourable prognosis in several cancer types, including colorectal cancer [3], head and neck squamous cell carcinoma (HNSCC), breast cancer, non-small-cell lung cancer (NSCLC) [1], and glioblastoma [4]. Clinically approved anti-EGFR monoclonal antibodies (cetuximab, panitumumab, and necitumumab) have been established as therapeutic [5,6]. In addition, small-molecule EGFR tyrosine kinase inhibitors (TKIs) have significantly improved the treatment of EGFR-mutant non-small-cell lung cancer, with multiple generations of inhibitors developed to target primary and resistance mutations [7]. In parallel, emerging modalities such as EGFR-targeted antibody–drug conjugates and near-infrared photoimmunotherapy represent promising next-generation strategies currently under preclinical and translational investigation [2,8].

While current clinical practice relies primarily on EGFR mutation status (e.g., exon 19 deletions or L858R in NSCLC, and KRAS/NRAS mutations in colorectal cancer) to guide targeted therapy, the predictive value of EGFR protein expression levels is still being actively investigated [9,10]. Higher EGFR expression, particularly as a composite index combining EGFR gene copy number (assessed by FISH) and protein expression (by IHC), correlates with improved overall survival when cetuximab is added to first-line chemotherapy in advanced NSCLC, suggesting a promising predictive biomarker [10,11,12]. Several investigations have also found a positive correlation between elevated EGFR expression, a high risk of prostate-specific antigen recurrence, and a trend toward androgen independence in prostate cancers [2].

The current clinical standard for assessing EGFR expression in human tumours relies on biopsy sampling [13]. The invasive nature of this procedure limits the number of accessible tumour sites and increases the risk of false-negative results due to the spatial heterogeneity of receptor expression. The varying degrees of EGFR expression and disparities in expression between primary tumours and metastases [14,15], the sensitivity and specificity of antibodies [16], and the changes in EGFR expression during therapy [17] pose challenges for accurately determining EGFR expression in disseminated disease. Non-invasive alternatives such as liquid biopsies (e.g., circulating tumour DNA, circulating tumour cells) have emerged for monitoring tumour genetics, but they primarily reflect genomic alterations rather than protein expression levels, and their sensitivity can be compromised by low tumour shedding or expression heterogeneity [18,19,20]. Thus, a robust methodology for whole-body evaluation of EGFR protein expression in metastatic sites remains essential [20,21].

Radionuclide molecular imaging provides a non-invasive, whole-body approach to cancer diagnosis and patient stratification for targeted therapy, directly addressing the limitations of biopsy with respect to invasiveness and tumour heterogeneity [22], while offering the unique advantage of visualising receptor expression in all lesions irrespective of tumour shedding, thereby overcoming the spatial and temporal limitations inherent to both tissue biopsies and liquid-based assays [23,24].

The need for sensitive, whole-body detection of EGFR expression has driven the development of radiolabelled imaging probes based on monoclonal antibodies [21,25], antibody fragments [26,27], affibody molecules [28,29] and scaffold proteins [30], and peptide-based ligands [31,32]. Compared with labelled antibodies, smaller agents such as F(ab′)_2_ fragments, single domain antibodies (sdAbs), and affibody molecules have demonstrated significant promise in preclinical studies, providing favourable tumour-to-background contrast at earlier imaging time points [33]. In addition to technetium-99m, the ^123^I (T_1/2_ = 13.27 h) is a suitable radionuclide in clinical single-photon emission computed tomography (SPECT) [34,35].

The small size of scaffold proteins (4–20 kDa), well below the glomerular filtration threshold of approximately 60 kDa, facilitates rapid tumour accumulation and efficient renal clearance of unbound tracer, thereby reducing background activity and enhancing imaging contrast [4,22,36,37]. It has to be noted that the rapid clearance from blood is also associated with lower tumour uptake compared with full-length antibodies. Despite the advantage of small size, developing appropriate labelling chemistries for scaffold proteins is also an essential prerequisite for high-contrast molecular imaging. Development of labelling chemistry includes selecting radionuclides and chelators or bifunctional linkers to conjugate to tumour-targeting proteins.

Designed ankyrin repeat proteins (DARPins) are small engineered scaffold proteins consisting of 4–6 ankyrin repeats that form a stable solenoid structure, with capping repeats at both ends. DARPins are specifically designed for high-affinity interaction with a broad spectrum of cancer-associated targets [38]. DARPin E01, developed using phage display methodology, binds to EGFR ectodomain III with an apparent equilibrium dissociation constant (K_D_) of 0.5 nM [39]. The epitope of this DARPin overlaps with the epitope of the natural ligand, EGF [4,39]. It was demonstrated that DARPin E01 inhibits EGFR phosphorylation [39]. Given this, protein imaging should be used at low doses so that systemic EGFR inhibition and associated problems are minimal. The high specificity and affinity make E01 a potential vector for radionuclide imaging of EGFR. Our previous investigation demonstrated that the use of radiolabelled DARPin E01 provided specific visualisation of EGFR expression in preclinical models [40]. Compared with existing mAb-based EGFR imaging agents, the DARPin E01 probe showed lower absolute tumour uptake but a comparable tumour-to-blood ratio, offering logistical advantages (imaging at 4 h vs. 2–4 days) [41]. Anti-EGFR affibodies show higher uptake and tumour-to-organ ratios. However, these affibodies are the product of years of optimisation, while the evaluation and optimisation of radiolabelled E01 was only initiated.

Our previous study demonstrated that the use of the ^123^I label offers an appreciable advantage compared with ^99m^Tc in the development of a probe for single-photon emission computed tomography (SPECT). The combination of the non-residualising label [^123^I]I-para-iodobenzoate (^123^I-PIB) and DARPin E01 resulted in low accumulation of activity in normal organs. The non-residualising ^123^I-PIB showed reduced hepatic retention of activity, resulting in the liver uptake being lower than tumour uptake. Usually, non-residualising labels contribute to a decreased cellular retention of activity after internalisation. Such an effect was not observed when DARPin E01 was labelled with a residualising label (^99m^Tc) [40]. After internalisation, a residualising label enables prolonged retention of a radionuclide in both tumour and normal tissues. Thus, using non-residualising labels might be appropriate for radiolabelled proteins that are internalised slowly in tumours but rapidly in normal organs and tissues. Previous investigations found that labelled DARPins, including HER2-binding DARPin G3 [36], EpCAM-binding Ec1 [30,42], and EGFR-binding E01 [40], were slowly internalised in cancer cells.

Radioiodination of targeting proteins, including DARPins, can be achieved via two principal strategies: direct labelling and indirect radioiodination using prosthetic groups. Direct radioiodination results in the coupling of radioiodine to tyrosine side-chains. Intracellular proteolysis produces radiometabolites—primarily iodide and iodotyrosine—that are recognised by enzymes and transporters involved in thyroid hormone metabolism, leading to elevated accumulation of radioactivity in Na/I-symporter-expressing organs and prolonged blood-pool retention [43]. An alternative method for introducing a radioiodine label with rapid excretion of its radiometabolites is indirect radioiodination using a prosthetic group. In this case, the accumulation of radiocatabolites in organs that include Na/I-symporters is typically lower than that of catabolites in directly iodinated proteins. N-succinimidyl-para-iodobenzoate (SPIB) has been successfully used as a prosthetic group for labelling monoclonal antibodies [44,45], affibodies and DARPins [40]. An alternative prosthetic group for radioiodine-labelling proteins is ((4-hydroxyphenyl)ethyl)maleimide (HPEM). HPEM enables site-specific attachment of labels and enables control of the location and quantity of labels per protein.

An obstacle to imaging EGFR is its strong expression in hepatocytes. The selection of a label and the method used for labelling might impact the distribution of the labelled protein within the body and the transport of radiocatabolites.

A possible disadvantage of [^123^I]I-(HE)_3_-E01-PIB is that this radiolabelling results in conjugation with [^123^I]I-para-iodobenzoate randomly attached to the eight lysine amino groups in DARPin (HE)_3_-E01. It is difficult to control the location and quantity of labels on each protein. Site-specific labelling with an HPEM bifunctional linker might overcome this problem. In the case of site-specific labelling using HPEM, cysteine must be introduced by gene engineering at the N- or C-terminus of DARPin for maleimide–thiol coupling. The DARPin scaffold does not contain cysteine. By adding a single cysteine, a precise control over the position and number of the label is possible. Lindbo S. et al. (2018) reported that the use of the [^125^I]I-HPEM label for another modified scaffold protein, ADAPT, provided very low retention of the renal radioactivity [46]. DARPin Ec1 variants labelled with [^125^I]I-HPEM at the C-terminus had low retention of activity in lungs, liver, spleen, kidneys, muscles, and bones, indicating a non-residualising character of the [^125^I]I-HPEM label [42]. Another study revealed that the incorporation of the neutral triglycyl- (GGG) amino acid sequence and the HPEM linker at the C-terminus of DARPin G3 resulted in increased local hydrophobicity of the conjugate, leading to the predominant excretion of the labelled protein through the hepatobiliary route [15]. Thus, it cannot be generalised that using HPEM has the same effect on biodistribution for all engineered scaffold proteins. Although the G3-HPEM conjugate is known to be cleared via the hepatobiliary route, systematic studies of E01-HPEM are needed to confirm whether this is a general phenomenon or a G3-specific feature.

The composition of cysteine-containing peptide-based moieties influences the biodistribution of labelled proteins. The use of negatively charged hydrophilic glutamate (residualising label) in cysteine-containing peptide-based chelators for ^99m^Tc reduced hepatic uptake of affibody molecules [33]. However, this effect might be counterbalanced by increased intracellular retention of a radionuclide in the hepatocytes [33]. The glycine-containing variant (non-residualising label) does not remain in cells because it diffuses away when the targeting proteins are degraded by proteolysis in lysosomal compartments. This results in a decrease in the accumulation of ^99m^Tc in the kidneys and liver [33]. Thus, the selection of an optimal cysteine-containing peptide-based labelling moiety might affect the distribution of the labelled protein within the body and the transport of radiocatabolites. Comprehensive investigations addressing the impact of labelling chemistry on the imaging attributes of radiopharmaceutical tracers utilising engineered scaffold proteins are essential to ensure heightened imaging sensitivity.

To evaluate the influence of the composition of cysteine-containing peptide-based linkers on the distribution of ^123^I-labelled DARPin E01, we investigated variants with the ^123^I-HPEM label connected with the C-terminus of DARPin E01 via a Glu-Glu-Glu-Cys linker (designated as [^123^I]I-E01-E_3_C-HPEM) and a Gly-Gly-Gly-Cys linker (designated as [^123^I]I-E01-G_3_C-HPEM). We hypothesised that site-specific conjugation of the non-residualising [^123^I]I-HPEM label via a hydrophilic E_3_C linker would significantly reduce hepatic retention and renal reabsorption compared to the G_3_C linker and the randomly conjugated PIB label, thereby achieving superior imaging contrast and a more favourable safety profile, while preserving high affinity to EGFR. We evaluated the affinity, cellular processing, biodistribution, and in vivo targeting properties of [^123^I]I-E01-E_3_C-HPEM and [^123^I]I-E01-G_3_C-HPEM, and compared these properties with those of the previous variant, [^123^I]I-(HE)_3_-E01-PIB.

## 2. Results

### 2.1. Protein Production and Characterisation

Two new DARPin E01 variants containing amino acid sequences Glu-Glu-Glu-Cys (E_3_C) and Gly-Gly-Gly-Cys (G_3_C) at the C-termini, as well as DARPin (HE)_3_-E01, were obtained using the previously published methodology [40]. The purity of all DARPin E01 variants exceeded 95%, as determined by gel electrophoresis (SDS PAGE) (Figure S1).

The amino acid sequences of the new DARPin E01 variants were determined by LC-MS/MS analysis, which included protein cleavage into peptide fragments, peptide separation, and identification under reproducible conditions. The proteins were pre-reduced with TCEP to disrupt disulphide bonds and then carbamidomethylated. Hydrolysis was performed by incubation with trypsin at 37 °C for 16 h. Using this method, 1228 (E01-G_3_C) and 1041 (E01-E_3_C) peptides were identified. It was established that the amino acid sequence overlap between the analysed proteins and the identified peptides was 100% (Figures S2 and S3).

### 2.2. Radiolabelling

The ((4-hydroxyphenyl)ethyl)maleimide spe(HPEM) was synthesised from tyramine and maleic anhydride by AcOH-assisted condensation [47] and purified by column chromatography on silica with a yield of 60%. The chemical purity was confirmed by 1H and 13C NMR spectroscopy (Figures S4 and S5 in Supplementary Materials).

**Figure 1:**
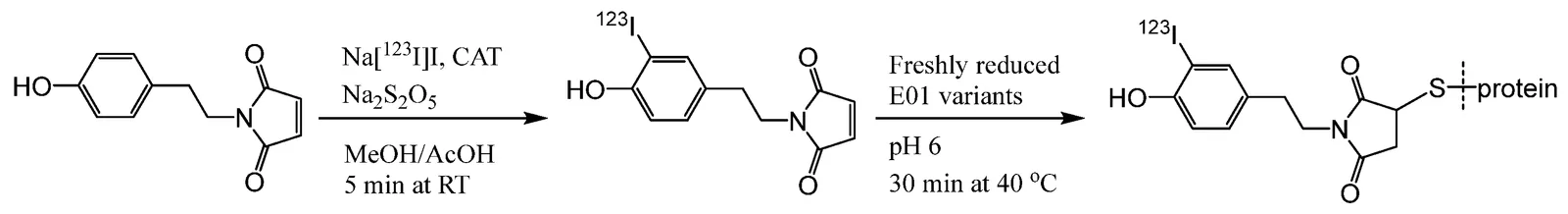
Site-specific radioiodination of [^123^I]I-E01-E_3_C-HPEM and [^123^I]I-E01-G_3_C-HPEM.

DARPin E01 variants with two different cysteine-containing linkers were labelled site-specifically by ^123^I through two steps. First, the prosthetic group HPEM with an activated phenolic ring and a maleimide group was iodinated with ^123^I. Thereafter, the ^123^I-HPEM was conjugated to DARPin E01 variants using maleimide–thiol coupling at the C-terminus of DARPin E01. This indirect labelling was performed as a one-pot, two-step procedure without intermediate purification. This approach reduces the handling of radioactive materials, resulting in lower radiation exposure for staff, reduced loss of labelled precursor during transfer, and a decreased risk of human error. It might result in lower specific activity, but this is not an issue for EGFR imaging probes [28]. The scheme of site-specific radioiodination of DARPin E01 variants is presented in Figure 1.

The [^123^I]I-E01-E_3_C-HPEM variant showed the lowest radiochemical yield (6 ± 2%) with a maximum apparent specific activity of 0.17 MBq/µg. The radiochemical yield of [^123^I]I-E01-G_3_C-HPEM was 13 ± 5% with a maximum specific activity of 0.28 MBq/µg. Site-unspecifically labelled DARPin (HE)_3_-E01 ([^123^I]I-(HE)_3_-E01-PIB) was obtained by attaching the N-hydroxysuccinimide ester derivative of ^123^I-PIB to the amino groups of lysines of DARPin (HE)_3_-E01, as previously reported [40]. Purification using the NAP-5 size-exclusion column yielded radiolabelled protein variants with radiochemical purities exceeding 98%. The characteristics of the labelling procedures and labelled DARPins are presented in Table 1.

### 2.3. In Vitro Studies

#### 2.3.1. Specificity Test

**Figure 2:**
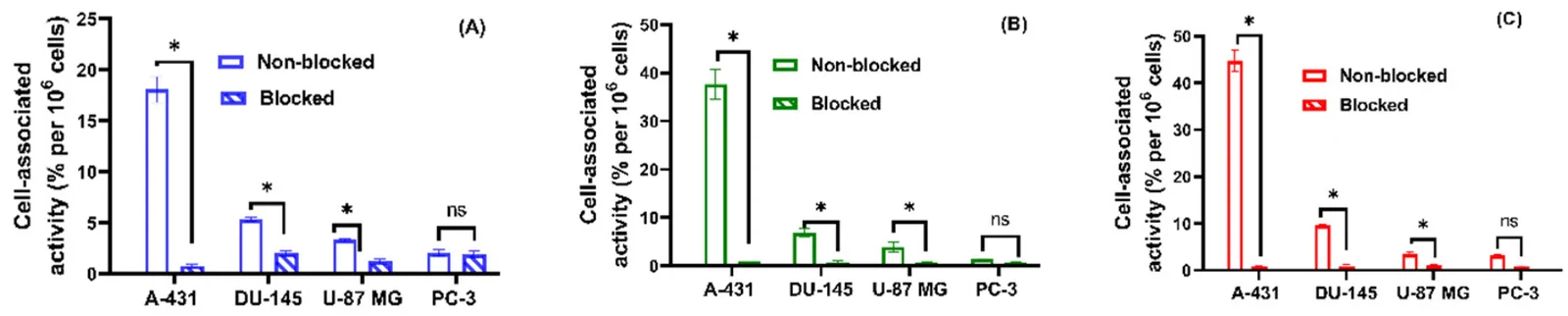
Binding specificity of (**A**) [^123^I]I-(HE)_3_-E01-PIB, (**B**) [^123^I]I-E01-E_3_C-HPEM, and (**C**) [^123^I]I-E01-G_3_C-HPEM to EGFR-expressing A-431, U-87 MG, DU-145 and PC-3 cells. For blocking, the cells were pre-incubated with a 100-fold molar excess of unlabelled cetuximab for 30 min at 37 °C before the addition of 5 nM radiolabelled DARPin E01 variants. The data are presented as the mean ± standard deviation (SD) of three samples. The symbol “*” means that the differences between groups are significant (*p* < 0.05, unpaired *t*-test). The symbol “ns” means that the differences between groups are not significant (*p* < 0.05, unpaired *t*-test).

The binding specificity of [^123^I]I-(HE)_3_-E01-PIB, [^123^I]I-E01-E_3_C-HPEM, and [^123^I]I-E01-G_3_C-HPEM was evaluated using cancer cell lines possessing different levels of EGFR expression, A-431, U-87 MG, DU-145, and PC-3. The results of binding specificity tests are presented in Figure 2. The levels of cell-bound activity depend on the level of EGFR expression and were highest for A-431 (~1.2 × 10^6^ receptors/cell) [48] as compared to DU-145 (~2 × 10^5^ receptors/cell) [48], U-87 MG (~1.0 × 10^5^ receptors/cell) [49], and PC-3 (~3 × 10^4^ receptors/cell) [48]. All radiolabelled E01 variants provided similar levels of cell-bound activity on the high-EGFR-expressing A-431 cell line (~18%). Pre-saturation treatment of EGFR with a molar excess of unlabelled cetuximab for all radiolabelled E01 variants resulted in a significant (*p* < 0.05, unpaired *t*-test) reduction in cell-associated radioactivity in all cells tested, except for PC-3, which has a very low EGFR expression level. Saturated binding characteristics of all radiolabelled E01 variants indicated EGFR-mediated specificity.

#### 2.3.2. Cellular Processing

**Figure 3:**
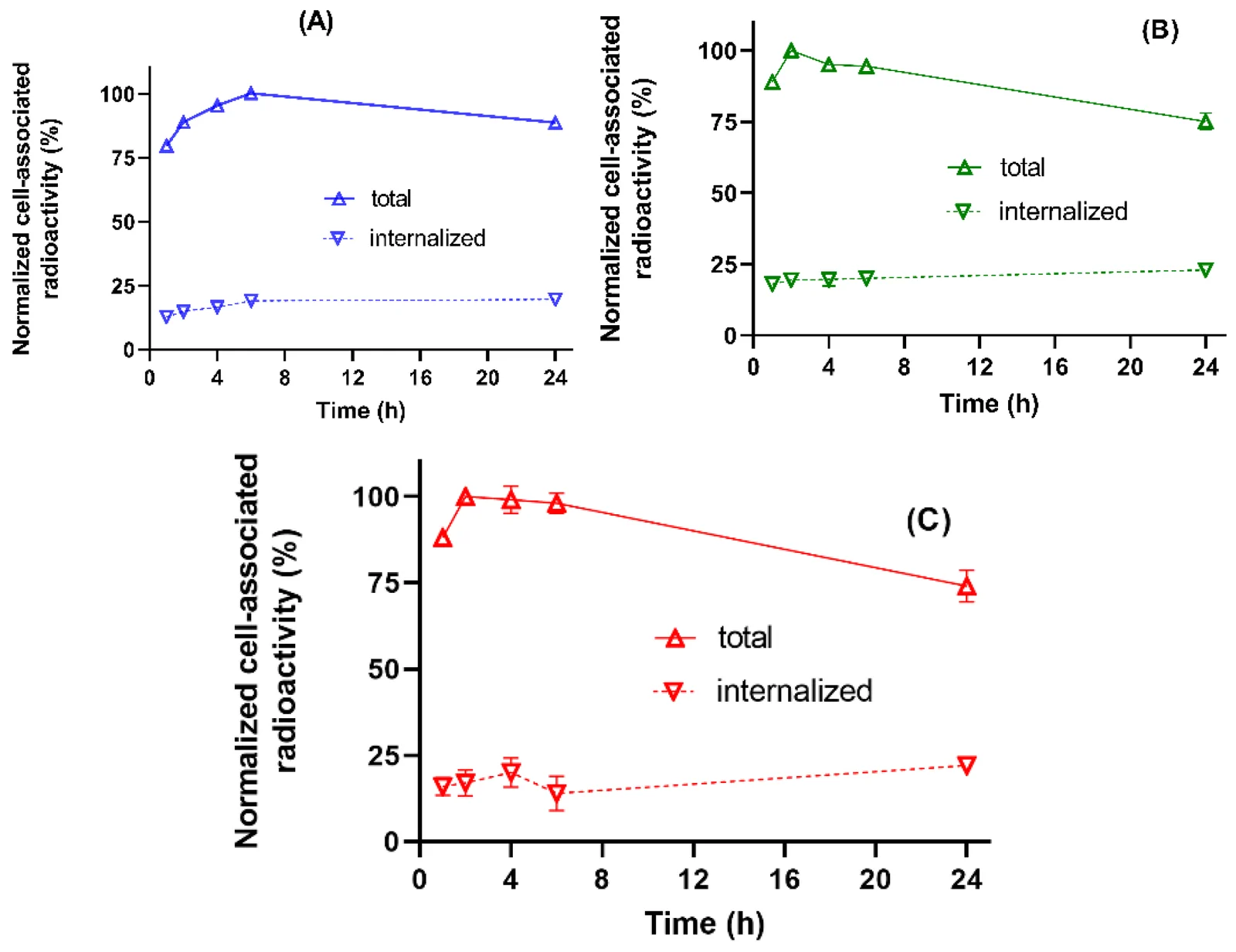
Cellular processing of (**A**) [^123^I]I-(HE)_3_-E01-PIB, (**B**) [^123^I]I-E01-E_3_C-HPEM, and (**C**) [^123^I]I-E01-G_3_C-HPEM in EGFR-expressing A-431 cells during continuous incubation at 37 °C. Data are presented as the mean of three samples ± SD. Error bars might not be visible when they are smaller than the symbols.

The processing of three radioiodinated DARPin E01 variants by EGFR-expressing A-431 cells was studied during continuous incubation for 24 h. The results of the processing assays are presented in Figure 3. Common features for the three radioiodinated DARPin E01 variants were rapid binding and a relatively low internalisation fraction. Total cell-associated activity increased up to 6 h of incubation, then decreased by 24 h (to approximately 75% of the maximum level). The decrease in cell-associated activity observed after 6 h could be explained by the non-residualising properties of the radioiodine labels, resulting in a diffusion of radiocatabolites from cells after internalisation. All variants showed the same level of internalised fractions at 24 h.

#### 2.3.3. Saturation Assay

**Figure 4:**
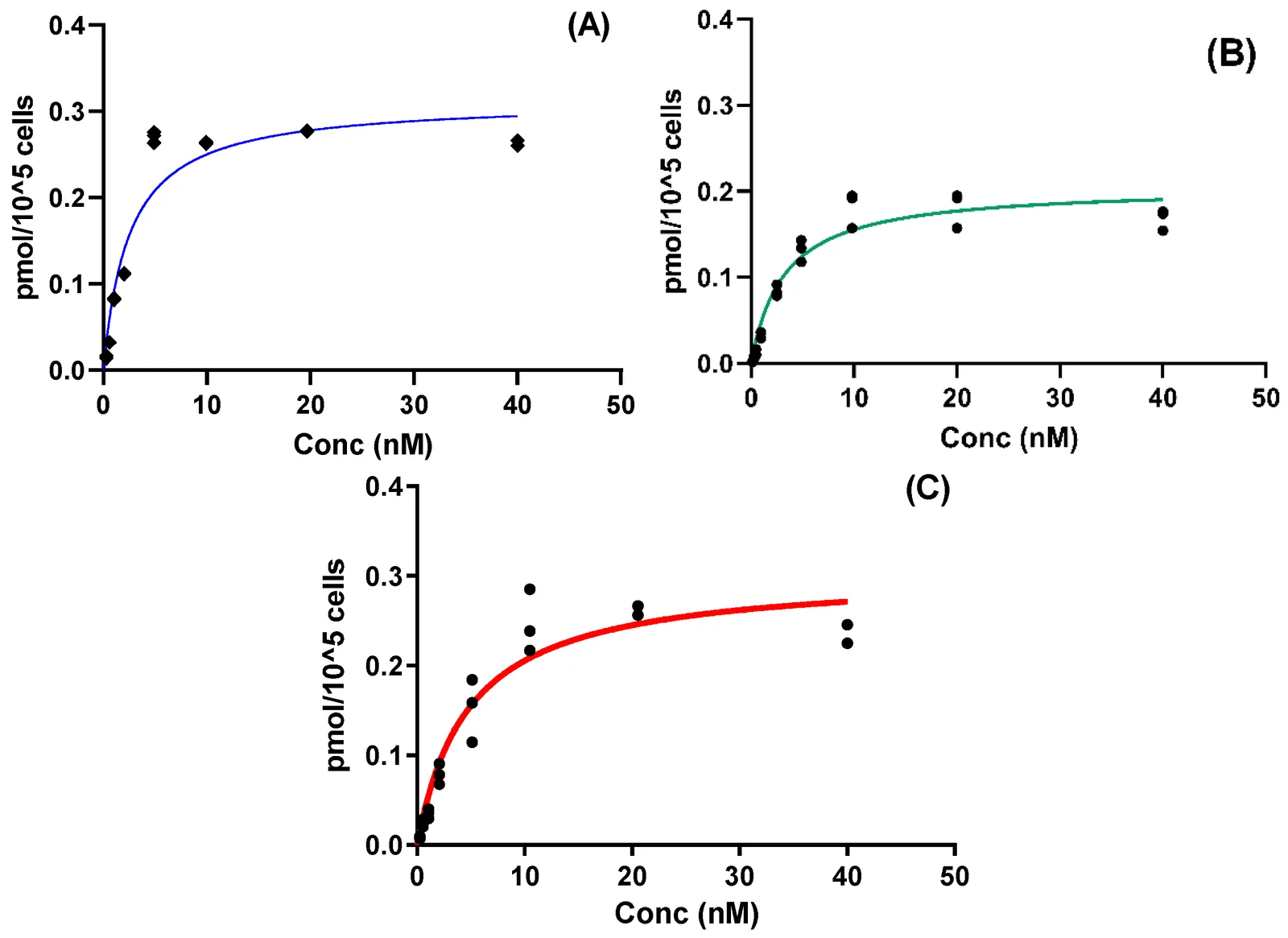
Determination of equilibrium dissociation constants (K_D_) of (**A**) [^123^I]I-(HE)_3_-E01-PIB, (**B**) [^123^I]I-E01-E_3_C-HPEM, and (**C**) [^123^I]I-E01-G_3_C-HPEM interaction with EGFR-expressing A-431 cells in vitro. The data are presented as the average value from three samples for each concentration.

The results of the binding affinity measurement of radioiodinated DARPin E01 variants to EGFR using A-431 cells are presented in Table 2 and Figure 4. The dissociation equilibrium constants (K_D_) and number of binding sites (B_max_) values were derived from the specific binding curves. The K_D_ values of all radioiodinated DARPin E01 variants for EGFR on A-431 cells were in the low nanomolar range, ranging from 2.7 to 4.8 nM. The variants with glutamate-containing linker ([^123^I]I-(HE)_3_-E01-PIB and [^123^I]I-E01-E_3_C-HPEM) showed lower K_D_ values than that of the variant with glycine-containing linker ([^123^I]I-E01-G_3_C-HPEM). The B_max_ of EGFR on A-431 cells was determined to be (1.10–1.66) × 10^6^ receptor sites per cell.

### 2.4. In Vivo Studies

**Figure 5:**
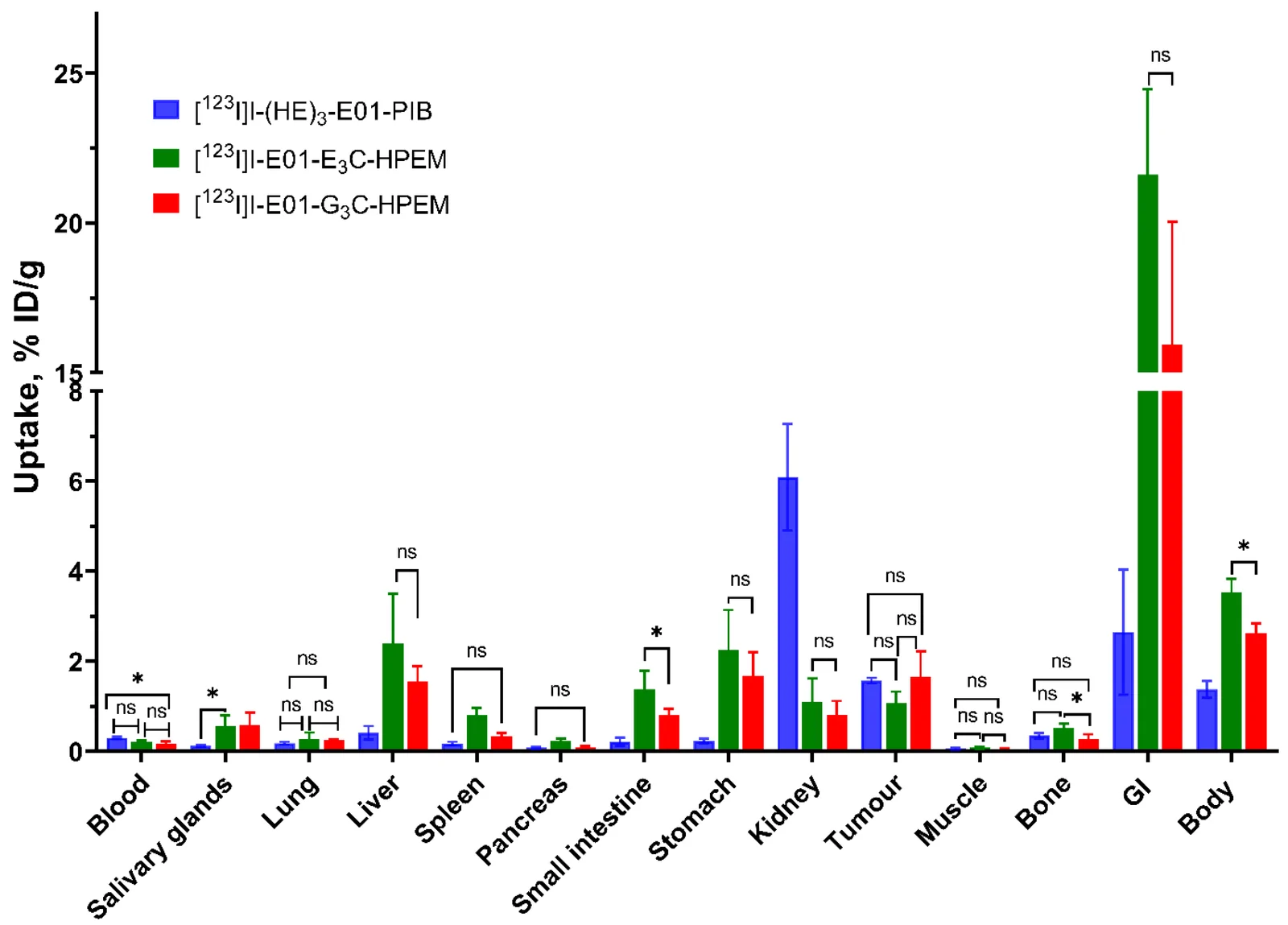
Comparative biodistribution of radiolabelled E01 variants at 4 h p.i. in Nu/J mice bearing EGFR-expressing A-431. Data are presented as mean ± SD for four mice. Data for the rest of the GI tract, its contents, and the rest of the body are presented as %ID per whole sample. The symbol “*” means that the differences between groups are significant (*p* < 0.05, ANOVA). The symbol “ns” means that the differences between groups are not significant (*p* < 0.05, ANOVA).

**Figure 6:**
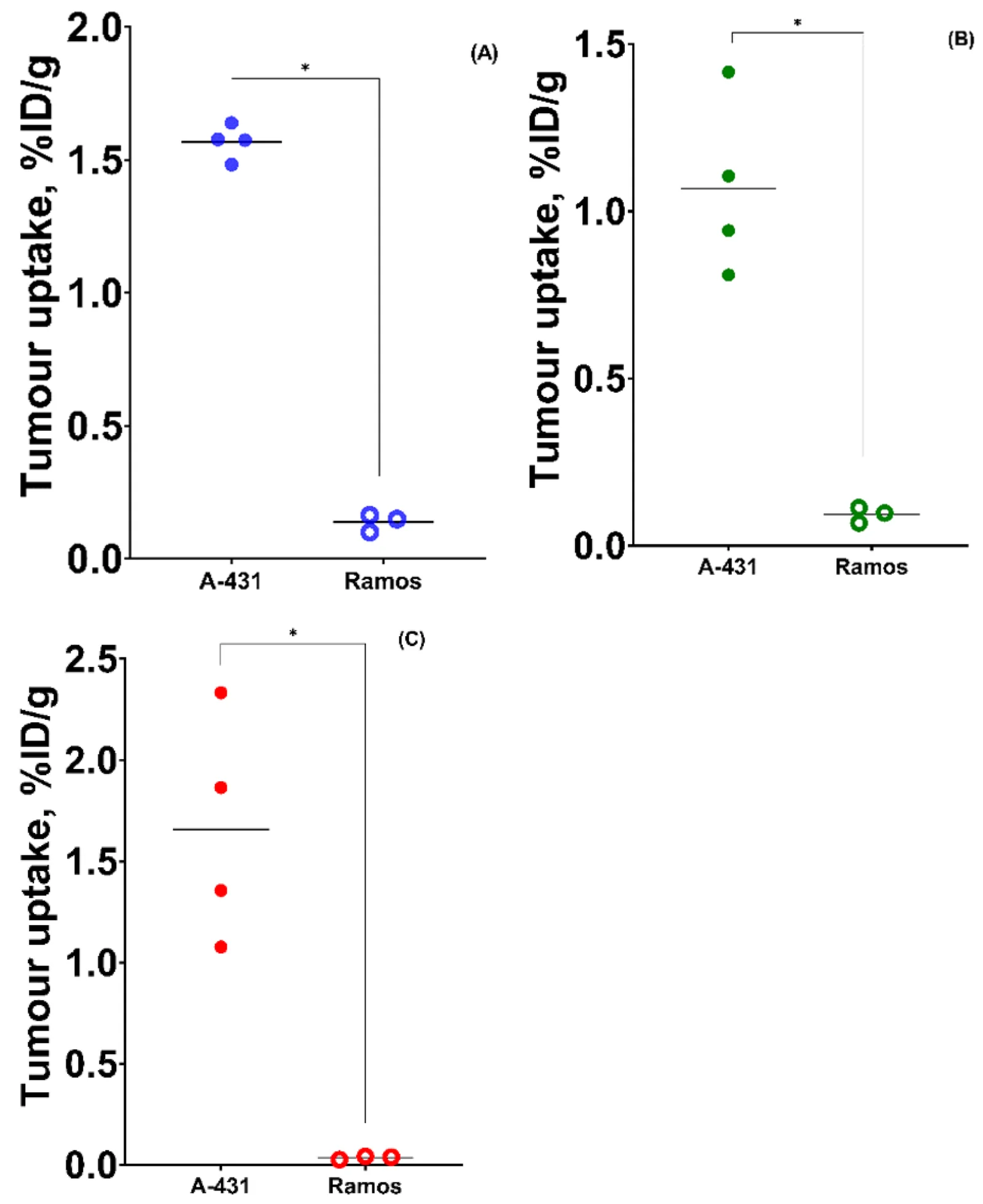
EGFR-specificity of tumour uptake of [^123^I]I-(HE)_3_-E01-PIB (**A**), [^123^I]I-E01-E_3_C-HPEM (**B**), and [^123^I]I-E01-G_3_C-HPEM (**C**) in Nu/J mice bearing EGFR-expressing A-431 and non-EGFR-expressing Ramos xenografts at 4 h p.i. Data are presented as the mean for four mice and individual values for each mouse. Asterisks indicate significant differences (*p* < 0.05, unpaired *t*-test).

A comparison of the biodistribution of novel radioiodinated DARPin E01 variants with the biodistribution of [^123^I]I-(HE)_3_-E01-PIB in Nu/J mice bearing EGFR-expressing A-431 xenografts at 4 h p.i. is presented in Figure 5. To test the tumour binding specificity, the tumour uptake of novel radioiodinated DARPin E01 variants was compared in EGFR-expressing A-431 and non-EGFR-expressing Ramos xenografts at 4 h p.i. (Table S1 in Supplementary Materials). No significant differences (*p* > 0.05) in activity levels in normal organs or tissues were observed between the A-431 and Ramos xenografts for any of the radioiodinated DARPin E01 variants. The significant differences (*p* < 0.05) in tumour uptake by A-431 and Ramos xenografts were observed in novel radiolabelled E01 variants (Figure 6).

[^123^I]I-(HE)_3_-E01-PIB, [^123^I]I-E01-E_3_C-HPEM, and [^123^I]I-E01-G_3_C-HPEM showed quite similar biodistribution profiles characterised by low uptake levels (<0.3%) in the blood, lung, pancreas, and muscle (Figure 5). [^123^I]I-(HE)_3_-E01-PIB provided a significantly lower uptake in the accumulation of iodine radiocatabolites in organs with expression of Na/I-symporters. The uptake of [^123^I]I-(HE)_3_-E01-PIB in the salivary glands was ca. five times lower than that of [^123^I]I-E01-E_3_C-HPEM and [^123^I]I-E01-G_3_C-HPEM. In the stomach, the uptake of [^123^I]I-(HE)_3_-E01-PIB was ca. ten times lower than [^123^I]I-E01-E_3_C-HPEM and ca. seven times lower than [^123^I]I-E01-G_3_C-HPEM. It is likely that the metabolism of ^123^I-PIB results in the decreased release of free radioiodide.

The levels of radioactivity accumulation in bone and spleen were low for all radioiodinated DARPin E01 variants. Uptake of the spleen of the [^123^I]I-E01-E_3_C-HPEM was higher than that of other variants.

[^123^I]I-(HE)_3_-E01-PIB, [^123^I]I-E01-E_3_C-HPEM and [^123^I]I-E01-G_3_C-HPEM provided similar (*p* > 0.05, ANOVA test) levels of A-431-tumour uptake, 1.7 ± 0.1%, 1.1 ± 0.7%, and 1.7 ± 0.6%, respectively (Figure 6). However, the tumour uptake of [^123^I]I-(HE)_3_-E01-PIB was higher than that in all normal organs and tissues except for the kidney. In the case of [^123^I]I-E01-E_3_C-HPEM, the tumour uptake was comparable to the small intestine uptake, and lower than the liver and stomach uptake. For [^123^I]I-E01-G_3_C-HPEM, the tumour uptake was comparable to that of the stomach and liver and exceeded the uptake in other organs. The hepatic uptake of [^123^I]I-(HE)_3_-E01-PIB was ca. six times lower than that of [^123^I]I-E01-E_3_C-HPEM and ca. four times lower than the uptake of [^123^I]I-E01-G_3_C-HPEM. The renal uptake levels of [^123^I]I-E01-G_3_C-HPEM, [^123^I]I-E01-E_3_C-HPEM and [^123^I]I-(HE)_3_-E01-PIB were 0.8 ± 0.3%, 1.1 ± 0.5%, and 6.1 ± 1.2%. The radioactivity accumulation in the gastrointestinal tract for [^123^I]I-(HE)_3_-E01-PIB (2.6 ± 1.9%) was much lower than that of [^123^I]I-E01-G_3_C-HPEM (21.7 ± 2.8%) and [^123^I]I-E01-G_3_C-HPEM (15.9 ± 4.1%). The accumulation of radioiodine activity in the small intestines for [^123^I]I-(HE)_3_-E01-PIB was approximately 6.5 times and four times lower than that for [^123^I]I-E01-E_3_C-HPEM and [^123^I]I-E01-G_3_C-HPEM, respectively. The complete data of the biodistribution of all variants in Nu/j mice bearing HER1-expressing A-431 and Ramos xenografts are presented in Table S1 (in Supplementary Materials).

**Figure 7:**
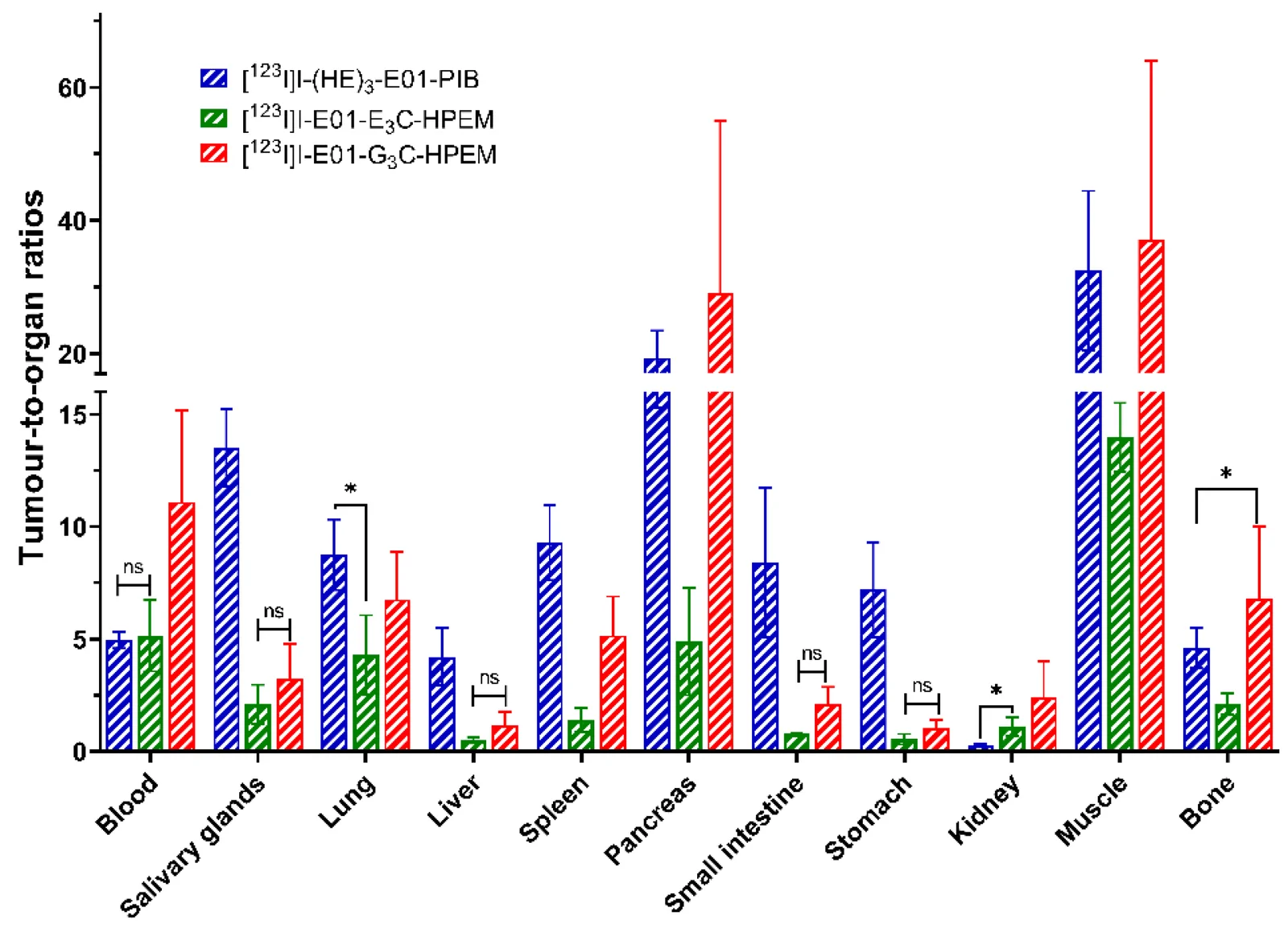
Comparison of tumour-to-organ ratios for radiolabelled E01 variants at 4 h p.i. in Nu/J mice bearing EGFR-expressing A-431 xenografts. Data are presented as the mean ± standard deviation from four mice. The symbol “*” means that the differences between groups are significant (*p* < 0.05, ANOVA). The symbol “ns” means that the differences between groups are not significant (*p* < 0.05, ANOVA).

[^123^I]I-(HE)_3_-E01-PIB showed the highest tumour-to-organ ratio for the majority of organs due to the low accumulation in the liver, small intestine, lung, spleen, and organs with an expression of Na/I-symporters (salivary gland and stomach). Although both [^123^I]I-E01-E_3_C-HPEM and [^123^I]I-E01-G_3_C-HPEM had good retention in tumours and provided similar tumour-to-organ ratios for some organs, there was a tendency to higher tumour-to-organ ratios for [^123^I]I-E01-G_3_C-HPEM (Figure 7). Moreover, [^123^I]I-E01-G_3_C-HPEM provided the highest tumour-to-organ ratios for blood, pancreas, and bone, compared to those of [^123^I]I-(HE)_3_-E01-PIB and [^123^I]I-E01-E_3_C-HPEM. The E01 variants labelled by [^123^I]I-HPEM with cysteine-containing peptide-based linkers provided a higher tumour-to-kidney ratio, but on the other hand, a lower ratio of tumour to liver and organs with expression of Na/I-symporters, compared to [^123^I]I-(HE)_3_-E01-PIB. The data of tumour-to-organ ratios for radioiodinated DARPin E01 variants at 4 h p.i. in Nu/J mice bearing EGFR-expressing A-431 xenografts are presented in Table S2 (in Supplementary Materials).

**Figure 8:**
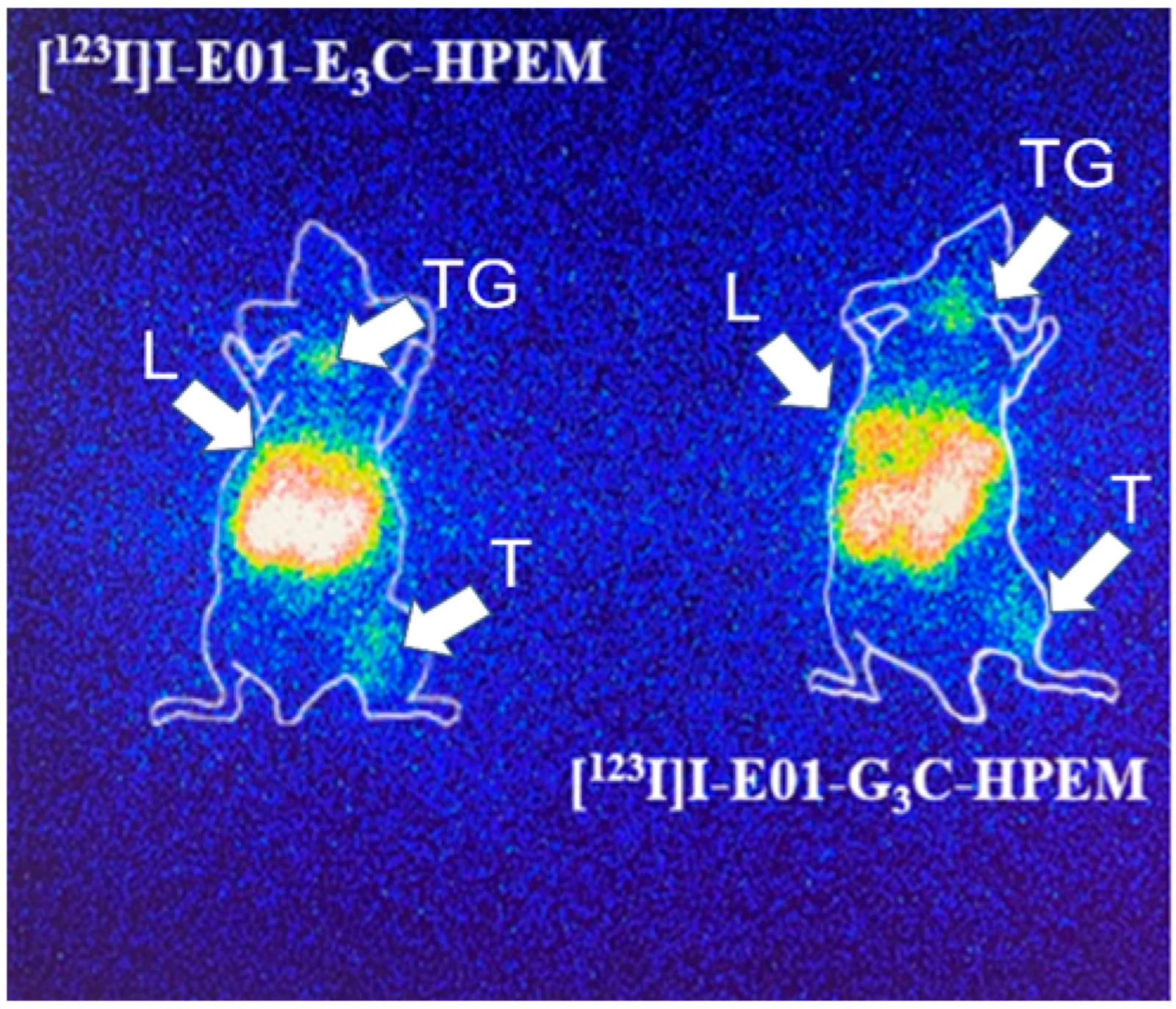
Gamma-camera imaging of EGFR expression in Nu/J mice bearing A-431 xenografts using [^123^I]I-E01-E_3_C-HPEM and [^123^I]I-E01-G_3_C-HPEM 4 h p.i. (3 µg, 0.9 MBq). T—tumour, L—liver, and TG—thyroid gland.

Gamma-camera imaging using [^123^I]I-E01-E_3_C-HPEM and [^123^I]I-E01-G_3_C-HPEM in Nu/J mice bearing A-431 xenografts at 4 h p.i. was performed to confirm the results of the biodistribution studies (Figure 8). Both variants visualised EGFR-expressing A-431 xenografts at 4 h p.i. The majority of the activity of both variants was eliminated from the blood at 4 h p.i. A high accumulation of activity was observed in other organs, such as the thyroid gland and liver. There also appeared to be a relatively high accumulation of stomach contents in the abdomen.

## 3. Discussion

EGFR overexpression is frequently associated with poor prognosis in several types of cancer. Radionuclide molecular imaging of cancer-related targets is considered a powerful, non-invasive tool for diagnosing and stratifying patients for targeted treatment. Nowadays, many studies show that small (5–20 kDa) scaffold protein-based tracers offer clear advantages for radionuclide imaging, including shorter time between injection and imaging and higher imaging contrast, compared with imaging probes based on full-length monoclonal antibodies [22,30,36]. However, more structure–property relationship information is necessary to enable customised design of scaffold protein-based tracers for specific applications. The scaffolds differ significantly in structure and composition, so knowledge about one scaffold might not directly translate to another. This study aims to assess the impact of labelling strategy on the imaging properties of radioiodinated DARPin E01 targeting EGFR expression. Our focus was on developing imaging probes for single-photon emission computed tomography (SPECT). Currently, positron emission tomography (PET) is superior in sensitivity and quantification to SPECT. However, SPECT is much more available globally. Thus, developing a SPECT-compatible imaging probe might have a significant impact on global healthcare [14].

When a prosthetic group of N-succinimidyl-*para*-iodobenzoate was used for indirect radioiodination of DARPin [40], there was concern that this might negatively affect its binding affinity for EGFR. The *para*-iodobenzoate might be attached to the amino group of the lysine residue in the EGFR-binding paratope. Furthermore, this method does not control the amount or position of the label within the protein. Indirect radioiodination using a maleimide-containing precursor would avoid such risk because a prosthetic group would be attached to a unique cysteine, providing a well-defined conjugate. In addition, the choice of a prosthetic group allows optimisation of biodistribution properties, including intracellular retention and excretion of radiocatabolites. So, this study tested the hypothesis that indirect radioiodination of DARPin using HPEM would provide a targeting profile favourable for SPECT imaging. In addition, the influence of the composition of cysteine-containing linkers at the C-terminus of DARPin E01 on its biodistribution and targeting properties was investigated. [^123^I]I-HPEM was conjugated to DARPin E01 at the C-terminus of DARPin E01 using two different linkers, a Glu-Glu-Glu-Cys linker ([^123^I]I-E01-E_3_C-HPEM) and a Gly-Gly-Gly-Cys linker ([^123^I]I-E01-G_3_C-HPEM). For comparison, a site-unspecifically radioiodinated DARPin E01 ([^123^I]I-(HE)_3_-E01-PIB) was produced by attaching the N-hydroxysuccinimide ester derivative of ^123^I-PIB to the amino groups of lysines, as previously reported by Larkina et al. [40].

At the first step, HPEM was radioiodinated using Chloramine-T with a high radiochemical yield, which is consistent with the data from a previous report [15]. However, the protein conjugation yields were low, which is typical for indirect radioiodination owing to competing hydrolysis during the coupling step. The [^123^I]I-E01-E_3_C-HPEM variant (with negatively charged glutamate in the linker at the C-terminus of DARPin) showed the lowest yield, possibly because glutamates closest to cysteine hinder deprotonation of the sulfhydryl group, reducing its reactivity. For clinical viability, these yields must be improved to >30–50%. However, variations in protein-to-prosthetic group ratio, temperature, and incubation time did not provide a significant yield increase in this study, indicating that more systematic optimisation might be required. It is worth noting that similar conditions for radioiodination of affibody Z(HER2:342) and DARPin G3-GGGC using HPEM enabled higher yields (45–51% and 31%, respectively) [36,50]. However, labelling of anti-EpCAM DARPin Ec1 using [^125^I]I-HPEM was much more efficient when cysteine was placed at the N-terminus (radiochemical yield 53 ± 1%) than at the C-terminus (radiochemical yield 19 ± 4%) [43], indicating that the order of the HPEM moiety relative to the protein influenced the efficiency of the labelling reaction [43]. Nevertheless, purification using NAP-5 columns enabled achieving of a radiochemical purity above 95%, which is compatible with in vivo testing.

E01 variants showed specific binding to the EGFR, and their binding levels were proportional to EGFR receptor levels in the tested cell lines (Figure 2). The pattern of cellular processing was similar for all radioiodinated DARPin E01 variants, with relatively low internalisation (Figure 3). Total cell-bound activity increased rapidly up to 6 h of incubation, then decreased slightly. This was most likely because the lipophilic radiometabolites, which were produced by intracellular proteolysis, diffuse from cells, thereby gradually reducing the total cell-bound activity. The binding affinity of radioiodinated DARPin E01 variants to EGFR on A-431 cells was in the low nanomolar range (Figure 4). The affinity of site-specifically labelled variants was not higher than the affinity of [^123^I]I-(HE)_3_-E01-PIB.

In vivo, the uptake of both [^123^I]I-E01-E_3_C-HPEM and [^123^I]I-E01-G_3_C-HPEM in EGFR-expressing A-431 was significantly higher (*p* < 0.05) than in non-EGFR-expressing Ramos xenografts. This indicated that the tumour uptake was dependent on EGFR expression in tumours, i.e., it was EGFR-specific.

A side-by-side comparison of all radioiodinated E01 variants revealed that all variants cleared rapidly from the blood, as expected for radiolabelled DARPins (Figure 5, Table S1). However, different linkers and prosthetic groups had a low effect on the accumulation of activity in the lung, pancreas, bone, and spleen. The major difference between the tested radioiodinated DARPin E01 variants at 4 h p.i. was the accumulation in the liver and kidneys. The hepatic uptake of [^123I^]I-(HE)_3_-E01-PIB was significantly lower than the uptake of [^123I^]I-E01-E_3_C-HPEM and [^123I^]I-E01-G_3_C-HPEM. Prior research demonstrated that including negatively charged amino acids at the N-terminus decreased the hepatic uptake of radiolabelled scaffold protein affibody [51]. Incorporation of an (HE)_3_ tag at the N-terminus decreased the liver uptake of ^99m^Tc-labelled HER2-binding DARPin G3 compared to variants containing hexahistidine tags [52]. This phenomenon was also observed when a glutamate-containing tag was introduced at the C-terminus of ^111^In-labelled DARPin G3 [53]. Thus, the low hepatic uptake of [^123I^]I-(HE)_3_-E01-PIB might be explained by the effect of the (HE)_3_ tag on the distribution of DARPin E01. In this study, we tested whether incorporating three glutamates into the linker at the C-terminus would also reduce hepatic uptake. Apparently, this was not the case, and the difference in the hepatic uptake of [^123I^]I-E01-E_3_C-HPEM and [^123I^]I-E01-G_3_C-HPEM was not significant. In addition, the hepatic uptake of these variants was significantly higher than the uptake of [^123I^]I-(HE)_3_-E01-PIB.

Meanwhile, the renal uptake of site-specifically labelled DARPin E01 variants was significantly lower compared to the uptake of [^123I^]I-(HE)_3_-E01-PIB. The renal uptake of [^123I^]I-E01-E_3_C-HPEM and [^123I^]I-E01-G_3_C-HPEM was similar. Most likely, this is caused by different rates of release of radiometabolites of [^123^I]I-HPEM and [^123^I]I-PIB labels. Multiple previous studies suggest that the level of renal reabsorption of labelled scaffold proteins is not affected by the label position or the chemical properties of the nuclide and chelator [42,54]. The reabsorption of proteins in the proximal renal tubules was also quick, and radiometabolism from non-residual radioiodine labels would diffuse from the cells. The radiometabolites of the [^123^I]I-HPEM label, iodo-((hydroxyphenyl)ethyl)maleimide-cysteine adduct, was more hydrophobic than the radiometabolites of the [^123^I]I-PIB label, iodobenzoate-lysine, so that the radiometabolites of the [^123^I]I-HPEM label exited the lysosomes and diffused from the kidney cells more quickly.

Apart from that, [^123I^]I-(HE)_3_-E01-PIB had a lower level of accumulated activity in organs with expression of Na/I-symporters (salivary gland, stomach, and thyroid). It appeared that the radiocatabolites formed after intracellular degradation of [^123I^]I-(HE)_3_-E01-PIB were less likely to accumulate in these organs with expression of Na/I-symporters and were efficiently excreted from the body.

The accumulation of activity in normal organs is very important because it affects high-contrast levels in molecular imaging. Although all radioiodinated DARPin E01 variants showed similar levels of tumour uptake, [^123I^]I-(HE)_3_-E01-PIB provided the highest tumour-to-organ ratios for most organs due to low accumulation in normal organs (salivary glands, lung, liver, small intestine, and stomach). Among variants that used a cysteine-containing bifunctional linker with HPEM as the prosthetic group, [^123I^]I-E01-G_3_C-HPEM showed a higher tumour-to-normal organ ratio. The tumour uptake of [^123I^]I-E01-G_3_C-HPEM also exceeded hepatic uptake.

To demonstrate the feasibility of SPECT imaging of EGFR-expressing xenografts using variants of a cysteine-containing bifunctional linker with HPEM as the prosthetic group, [^123I^]I-E01-E_3_C-HPEM and [^123I^]I-E01-G_3_C-HPEM were evaluated. For both variants, the EGFR-expressing A-431 xenografts were visualised, and the majority of the activity of both variants was eliminated from the blood at 4 h p.i. High activity accumulation was observed in other organs, such as the thyroid gland and liver. There was a significant accumulation of activity in the abdomen, which matched the substantial accumulation of both variants in the gastrointestinal tract and stomach, as observed in the in vivo biodistribution data. Previously, we have demonstrated that labelling of other scaffold proteins (affibody molecules and ADAPTs) using [^125^I]I-HPEM results in rapid excretion of radiometabolites, and low uptake in the salivary glands and stomach [33,46,50]. However, application of this strategy for the labelling of anti-HER2 DARPin G3 resulted in elevated hepatobiliary excretion of the conjugate, causing decreased tumour uptake and a high activity level in the contents of the gastrointestinal tract. Similarly, the biodistribution of anti-EpCAM DARPin Ec1 and anti-EGFR-DARPin E01 did not benefit from the HPEM strategy. The magnitude of the effect may vary depending on the net charge, size, and overall surface properties of a protein, as these influence recognition by transporters on hepatocytes and tubular reabsorption. In addition, the position of the label matters. This suggests that the effect of charged tags is position dependent, and the proximity of the HPEM moiety to the protein surface may also influence interactions with hepatic transporters.

Overall, our previous and current data show that HPEM has dissimilar effects on different scaffold proteins. HPEM conjugation was beneficial for biodistribution of affibody and ADAPT, but detrimental for DARPin variants. Collectively, these findings suggest that developing a radioiodinated DARPin E01-HPEM conjugate for EGFR imaging is not advisable. Variations in HPEM position and linker may not yield improvements. Further modifications are unlikely to succeed. This approach should be abandoned.

## 4. Materials and Methods

### 4.1. General Materials and Instruments

The concentration of purified DARPin E01 variants was determined in triplicate on a NanoDrop OneC microvolume UV-Vis spectrophotometer (Thermo Scientific, Waltham, MA, USA). [^123^I]NaI was obtained from the Tomsk Polytechnic University R-7M cyclotron facility by irradiating a [^122^Te]TeO_2_ target with 13.6 MeV deuterons with a current of 20 µA. Thin-layer chromatography (TLC) analysis was performed using an iTLC glass microfibre chromatography sheet impregnated with a silica gel (Agilent Technologies, Inc., Folsom, CA, USA). The percentage of free ^123^I and radioiodinated proteins was determined using a Minigita TLC scanner with Gina Star TLC software version 6.3 (Elysia Raytest, Straubenhardt, Germany). Radioiodinated proteins were purified using NAP-5 size-exclusion columns (Cytiva, Uppsala, Sweden). The activity distribution was measured using a thallium-activated sodium iodide (NaI(Tl)) detector using the automated gamma counter Wizard 2480 (Perkin Elmer, Waltham, MA, USA). The radioactivity of samples was measured by a dose calibrator (RIS-1A, Amplituda, Saint Petersburg, Russia). Whole-body gamma-camera imaging of mice was performed using a Siemens ECAM 180 scanner with a high-resolution, low-energy collimator (Siemens, Munich, Germany). Cells were cultured in a humidified incubator with 5% CO_2_ at 37 °C in Roswell Park Memorial Institute (RPMI) medium supplemented with 10% foetal bovine serum (FBS), 2 mM L-glutamine, 100 IU/mL penicillin, and 100 µg/mL streptomycin. In vitro experiments were performed in 35 mm Petri dishes (Nunc Delta Surface, Thermo Fisher Scientific, Roskilde, Denmark). The unpaired two-tailed *t*-test or ANOVA was used to determine whether there was a significant difference (GraphPad Software 8.0, San Diego, CA, USA).

### 4.2. Protein Production

The EGFR-specific DARPins (HE)_3_-E01, E01-G_3_C, and E01-E_3_C were produced in *E. coli* BL21(DE3) (Novagen-EMD Millipore, Madison, WI, USA) as *C*-terminal extensions of SUMO and purified as described earlier [40].

The variant (HE)_3_-E01 contains the HEHEHE ((HE)_3_) tag placed at the N-terminus. Its amino acid sequence is as follows:

MRGSHEHEHEGSDLGKKLLEAARAGQDDEVRILMANGADVNADDTWGWTPLHLAAYQGHLEIVEVLLKNGADVNAYDYIGWTPLHLAADGHLEIVEVLLKNGADVNASDYIGDTPLHLAAHNGHLEIVEVLLKHGADVNAQDKFGKTAFDISIDNGNEDLAEILQKLN

The variant E01-E_3_C had three glutamates followed by a cysteine at the *C*-terminus. Its amino acid sequence is as follows:

SDLGKKLLEAARAGQDDEVRILMANGADVNADDTWGWTPLHLAAYQGHLEIVEVLLKNGADVNAYDYIGWTPLHLAADGHLEIVEVLLKNGADVNASDYIGDTPLHLAAHNGHLEIVEVLLKHGADVNAQDKFGKTAFDISIDNGNEDLAEILQKLNGEEEC

The variant E01-G_3_C had three glycines followed by a cysteine at the *C*-terminus. Its amino acid sequence is as follows:

SDLGKKLLEAARAGQDDEVRILMANGADVNADDTWGWTPLHLAAYQGHLEIVEVLLKNGADVNAYDYIGWTPLHLAADGHLEIVEVLLKNGADVNASDYIGDTPLHLAAHNGHLEIVEVLLKHGADVNAQDKFGKTAFDISIDNGNEDLAEILQKLNGGGGC

The purified DARPins concentration was determined using a NanoDrop OneC (Thermo Fisher Scientific, Wilmington, DE, USA) using ε_280_.

Analysis of the E01-G_3_C and E01-E_3_C variants was performed using the Ultimate 3000 Nano LC System (Thermo Fisher Scientific, Waltham, MA, USA) and an Orbitrap Tribrid Lumos mass spectrometer (Thermo Fisher Scientific) equipped with a nanoelectrospray source (Thermo Fisher Scientific), following the methodology described earlier [47].

To ensure cysteine availability for conjugation, the DARPins E01-E_3_C and E01-G_3_C were reduced using dithiothreitol (DTT). The 150-fold molar excess of DTT (4.1 µL of 1 M solution; 632 µg; 4.1 nmol) was added to a solution of E01-E_3_C (1000 µg; 57 nmol) or E01-G_3_C (1000 µg; 57.8 nmol) in degassed PBS (230 µL). Following incubation at 40 °C for 1 h, E01-E_3_C or E01-G_3_C was purified using a NAP-5 size-exclusion column and pre-equilibrated with degassed 0.2 M NH_4_OAc (pH 6.0).

### 4.3. Synthesis of ((4-Hydroxyphenyl)ethyl)maleimide

((4-hydroxyphenyl)ethyl)maleimide (HPEM) was synthesised using a method adapted from a previously published method [55]. Firstly, tyramine (274 mg, 2.0 mmol) and maleic anhydride (220.5 mg, 1.9 mmol) were diluted in glacial acetic acid (3 mL). The mixture was refluxed at 120 °C for 1.5 h. The reaction mixture was then cooled to room temperature. H_2_O (15 mL) was added, and the aqueous phase was extracted several times with EtOAc. The organic layers were washed with 1 M NaHCO_3_. Afterward, the mixture was dried using Na_2_SO_4_ and concentrated under a vacuum to obtain the crude products. The resulting crude product was purified by column chromatography (from 100% CH_2_Cl_2_:0% Et_2_O to 90% CH_2_Cl_2_:10% Et_2_O) to give white crystals (60% yield). The ^1^H NMR and ^13^C NMR spectra of ((4-hydroxyphenyl)ethyl)maleimide are presented in Figures S4 and S5 (in Supplementary Materials). ^1^H NMR (CDCl_3_, 400 MHz) δ 6.99 (2H, dd, *J* = 8.5 Hz), 6.68 (2H, dd, *J* = 8.5 Hz), 6.58 (2H, s), 3.65 (2H, t, *J* = 7.5 Hz), 2.76 (2H, t, *J* = 7.7 Hz).

^13^C NMR (MeOD, 101 MHz) δ 170.95, 155.72, 133.88, 129.41, 128.78, 114.84, 38.87, 33.04.

### 4.4. Radiolabelling

In general, indirect labelling of DARPin E01 variants was performed in two steps: the radioiodination of the prosthetic group and then the conjugation of a radioiodinated prosthetic group to the protein.

Radiolabelling of DARPin E01 variants with two different cysteine-containing linkers. The first step was HPEM labelling using ^123^I. A [^123^I]NaI solution (200–300 µL, 150–300 MBq) was adjusted to pH 5–6 using 0.1% CH_3_COOH solution. Then, ((4-hydroxyphenyl)ethyl)maleimide (23 nmol, 5 µg, 5 µL of 1 mg/mL previously dissolved in CH_3_OH/CH_3_COOH; 95/5 (*v*/*v*)) was added to the [^123^I]NaI solution. Chloramine-T solution (40 µg, 10 µL, 4 mg/mL in water) was added to the reaction mixture to start the reaction. After 5 min at room temperature, sodium metabisulfite solution (60 µg, 10 µL, 6 mg/mL in water) was added to the reaction mixture. The radiochemical yield of [^123^I]I-HPEM was measured using radio-iTLC with ethyl acetate as a mobile phase. The second step was the conjugation of [^123^I]I-HPEM and DARPin E01 variants. DARPin-E01-E_3_C (6.39 nmol, 112 µg, 100 µL of 1.12 mg/mL in 0.2 M acetic ammonium buffer, pH 6.0) or DARPin-E01-G_3_C (6.47 nmol, 112 µg, 100 µL of 1.12 mg/mL in 0.2 M acetic ammonium buffer, pH 6.0) were added to the reaction mixture. After 30 min of incubation at 40 °C, the radiolabelled conjugate was purified by size-exclusion chromatography on disposable NAP-5 columns, pre-equilibrated with PBS, and eluted with PBS. The radiochemical yield and purity of DARPin-E01-E_3_C and DARPin-E01-G_3_C were measured using radio-iTLC analysis in an acetone:water (4:1) system.

Site-unspecifically indirect labelled DARPin (HE)_3_-E01 with iodine-123, ([^123^I]I-(HE)_3_-E01-PIB) was obtained by attaching the N-hydroxysuccinimide ester derivative of ^123^I-PIB to the amino groups of lysines in DARPin (HE)_3_-E01, as per the previously reported methodology in Larkina et al. [40].

### 4.5. In Vitro Studies

Four human cancer cell lines with different levels of EGFR expression, epidermoid carcinoma A-431 (~1.2 × 10^6^ receptors/cell), prostate adenocarcinoma DU-145 (~2 × 10^5^ receptors/cell), glioblastoma U-87 MG (~1.0 × 10^5^ receptors/cell), and prostate carcinoma PC-3 (~3 × 10^4^ receptors/cell) were used for in vitro studies. All cell lines were purchased from the American Type Culture Collection (ATCC, Manassas, VA, USA). The cells were seeded into culture dishes to obtain a density of 10^6^ cells/dish during the experiments.

To investigate the binding specificity of all radioiodinated DARPin E01 variants to the EGFR in A-431, DU-145, U-87 MG, and PC-3, the cells were seeded into culture dishes (two sets (six wells) of dishes were used for each cell line). To a set of control dishes, a 100-fold molar excess of unlabelled cetuximab was added 30 min before the radioiodinated DARPin E01 molecules were added to saturate EGFRs. Cells were incubated for 1 h at 37 °C with 5 nM radioiodinated DARPin E01 variants. After incubation, the medium was collected. The cells were washed with medium, treated with trypsin–EDTA solution per dish, incubated for 10 min at 37 °C, and collected. Cell radioactivity was measured with a gamma counter, and the percentage of cell-bound radioactivity was calculated. The experiments were performed in triplicate.

To study the cellular processing, all radioiodinated DARPin E01 variants were evaluated in high-EGFR-expressing A-431 cells. The cells were incubated with 5 nM radioiodinated DARPin E01 variants at 37 °C for 1, 2, 4, 6, and 24 h. The supernatant was collected at the predetermined time intervals. After that, the cells were washed with PBS and collected in the same tube as the supernatant. To collect the membrane-bound activity, the cells were treated with 1 mL PBS and incubated on ice for 5 min in 0.2 M glycine buffer, pH 2, containing 4 M urea. To measure the internalised activity, the cells were incubated at 37 °C for 30 min with 1 mL of 1 M NaOH; then, the cell debris was collected. An additional 1 mL of NaOH was used to wash the dish. The radioactivity of the samples was measured using a gamma counter.

A saturation assay was performed to determine the equilibrium dissociation constant (K_D_) of all radioiodinated DARPin E01 variants to living A-431 cells. All radioiodinated DARPin E01 variants with concentrations ranging from 0.20 to 40 nM were used to perform this assay. For each protein variant concentration tested, three dishes were used to investigate specific binding, and a single cell culture dish was used to assess nonspecific binding via receptor blocking. Following a 4 h incubation at 4 °C, the medium containing the radioactive solution was recovered from each dish. Subsequently, the dishes were rinsed with PBS five times. The cells were then trypsinised with 500 µL of trypsin–EDTA per dish (10 min at 37 °C), and 1 mL of medium was added to each dish. A 500 µL sample was collected and placed into a cell counter, while the remaining 1 mL was measured for radioactivity using a gamma counter.

### 4.6. Animal Studies

All animal studies were approved by the Ethics Committee of Siberian State Medical University, Tomsk, Russia (protocol code 2, 27 September 2022). Biodistribution of all radioiodinated E01 variants was studied in Nu/j mice bearing A-431 xenografts with EGFR expression. Nu/j mice bearing Ramos xenografts with negative EGFR expression were also used as a specificity control. Four mice with A-431 xenografts and three with Ramos xenografts were used per data point in the in vivo experiments. To establish cancer xenografts, 10^7^ cells/animal in 100 μL of medium were subcutaneously implanted into female Nu/j mice. The average weights of the animals at the experiment time were 26.3 ± 2.3 g in the A-431 group and 26.1 ± 1.1 g in the Ramos group. For each variant, two groups of mice were injected intravenously (via the tail vein) with a radiolabelled solution (3 µg, 40 kBq, 100 µL in PBS with 1% BSA). The biodistribution of all radioiodinated DARPin E01 variants was assayed 4 h p.i. The mice were anaesthetised by cervical dislocation, and then all organs of interest were collected, weighed, and measured for their activity content using the gamma counter. Activity uptake was calculated as the percentage of the injected dose per gram of sample (% ID/g).

To perform whole-body gamma-camera imaging, the Nu/j mice bearing EGFR or Ramos xenografts were injected with 3 µg, 900 kBq of [^123^I]I-E01-E_3_C-HPEM or [^123^I]I-E01-G_3_C-HPEM. Images were acquired within 30 min and stored as a 1024 × 256-pixel matrix. The contours of animals were derived from digital photographs and superimposed onto the gamma-camera image to enhance comprehension.

## 5. Conclusions

In conclusion, indirect site-specific radioiodination of DARPin E01 with [^123^I]I-HPEM using cysteine-containing peptide-based linkers yielded stable labelling of DARPin E01. The conjugates demonstrated preserved binding capacity to EGFR-expressing human cancer cells in vitro and specific accumulation in EGFR-expressing xenografts in vivo. However, an appreciable level of hepatobiliary excretion and activity in organs expressing Na/I-symporters was observed in vivo for [^123^I]I-E01-E_3_C-HPEM and [^123^I]I-E01-G_3_C-HPEM, which hampered efficient tumour targeting. Indirect radioiodination with [^123^I]I-PIB is therefore the preferred approach for labelling DARPin E01 for further clinical SPECT imaging of EGFR expression.

### 5.1. Strengths and Limitations of the Current Study

This study provided a systematic comparison of different labelling strategies for DARPin E01 protein to gain structure–property relationship information for selecting the best tracer for EGFR imaging. However, several limitations should be noted. The internalisation data were measured only qualitatively and did not yield formal numerical measurements (e.g., the proportion of internalised activity compared to surface-bound activity), limiting our interpretation of intracellular retention and transport. The number of animals per data point was relatively small. Binding to mouse EGFR was not assessed in this study, as the protein lacked affinity for the murine receptor. Given the expression of EGFR in hepatocytes, cross-reactivity would have been valuable for interpreting biodistribution and for selecting appropriate doses in this preclinical model. The in vivo targeting specificity was demonstrated by comparison with EGFR-negative tumours, but it might be further confirmed by in vivo blocking.

### 5.2. Future Studies

The results of this study suggest that the [^123^I]I-(HE)_3_-E01-PIB would be more advisable for further clinical development. Further steps should include in vivo toxicity evaluation and dosimetry estimation. The dosimetry would require measuring biodistribution at several time points, upscaling animal data to humans, and calculating absorbed dose using, e.g., OLINDA/EXM software version 1.1.

An intriguing question is why HPEM-mediated radioiodination provides favourable imaging properties for affibodies and ADAPTs [46,50] but not for DARPins. Better understanding of structure–activity relationships would promote development of radiolabelled scaffold proteins for radionuclide targeting.

The following studies could be envisioned. Molecular modelling or structural analysis would be valuable to elucidate why the G3C linker reduces receptor affinity and possibly to suggest alternative linker lengths and compositions of linkers for experimental evaluation.

Metabolite profiling in plasma, urine, and kidney homogenates using radio-HPLC would help to identify the actual circulating and excreted catabolites and to verify whether they are indeed non-residualising. It would be of interest to investigate whether the (HE)_3_ tag would reduce hepatic uptake when applied to HPEM-labelled variants, or optimise the existing PIB approach by controlling average labelling stoichiometry rather than pursuing HPEM modifications.

## Figures and Tables

**Table 1:** Characteristics of radiolabelled E01 variants.

Variant	Radiochemical Yield, %	Isolated Yield, %	Radiochemical Purity, %	Maximum Specific Activity, MBq/µg
[^123^I]I-HPEM	86 ± 10	n/a	n/a	89.6
[^123^I]I-E01-E_3_C-HPEM	6 ± 2	5 ± 2	98 ± 2	0.17
[^123^I]I-E01-G_3_C-HPEM	13 ± 5	10 ± 2	98 ± 2	0.28
[^123^I]I-PIB	84 ± 1	n/a	n/a	25
[^123^I]I-(HE)_3_-E01-PIB	22 ± 1	18 ± 1	100 ± 0	0.17

**Table 2:** K_D_ and B_max_ values for the interaction between radioiodinated DARPin E01 variants and A-431 cells.

Parameters	[^123^I]I-(HE)_3_-E01-PIB	[^123^I]I-E01-E_3_C-HPEM	[^123^I]I-E01-G_3_C-HPEM
K_D_, nmol	2.7 ± 0.5	3.2 ± 0.6	4.8 ± 0.9
B_max_, receptors/cell	(1.66 ± 0.70) × 10^6^	(1.10 ± 0.73) × 10^6^	(1.43 ± 0.71) × 10^6^

## Data Availability

The original contributions presented in this study are included in the article/Supplementary Materials. Further inquiries can be directed to the corresponding author.

## Supplementary Materials

The following supporting information can be downloaded at https://www.mdpi.com/article/10.3390/ijms27167438/s1.
