# Supplementary material for: Evaluation of Site-Specific Radioiodination of Anti-EGFR-Targeted DARPin E01 Using (4-Hydroxyphenyl)ethyl Maleimide
Source: Int J Mol Sci. 2026 Aug 20;27(16):7438. doi: 10.3390/ijms27167438 (PMC13513505; doi:10.3390/ijms27167438)
Supplement: Supplementary file 1 [file ijms-27-07438-s001.zip › ijms-4442993-supplementary.pdf]

Supplementary Materials

Evaluation of Site-Specific Radioiodination of Anti-EGFR-Targeted DARPin E01 using (4-hydroxyphenyl)ethyl maleimide

Mariia Larkina [1,2], Gleb Yanovich [1,2], Lutfi A. Hasnowo [3], Ruslan Varvashenya [1], Daria Eskova[1], Ivan Sharychev [1], Anastasia Prach [1], Evgenii Plotnikov [1], Roman Zelchan [1,4], Alexey Schulga [1,5], Elena Konovalova [1,5], Rustam H. Ziganshin [6], Mikhail Belousov [1,2], Vladimir Tolmachev [7,*] and Sergey M. Deyev [1,5]

1 Research Centrum for Oncotheranostics, Research School of Chemistry and Applied Biomedical Sciences, Tomsk Polytechnic University, 634050 Tomsk, Russia
2 Department of Pharmaceutical Analysis, Siberian State Medical University, 634050 Tomsk, Russia
3 Polytechnic Institute of Nuclear Technology, National Research and Innovation Agency of Indonesia, Yogyakarta 55281, Indonesia
4 Department of Nuclear Medicine, Cancer Research Institute, Tomsk National Research Medical Center, Russian Academy of Sciences, 634009 Tomsk, Russia
5 Molecular Immunology Laboratory, Shemyakin & Ovchinnikov Institute of Bioorganic Chemistry, Russian Academy of Sciences, 117997 Moscow, Russia
6 Collective Use Center "Bioorganic", Shemyakin & Ovchinnikov Institute of Bioorganic Chemistry, Russian Academy of Sciences, 117997 Moscow, Russia
7 Department of Immunology, Genetics and Pathology, Uppsala University, 75185 Uppsala, Sweden
* Correspondence: vladimir.tolmachev@igp.uu.se

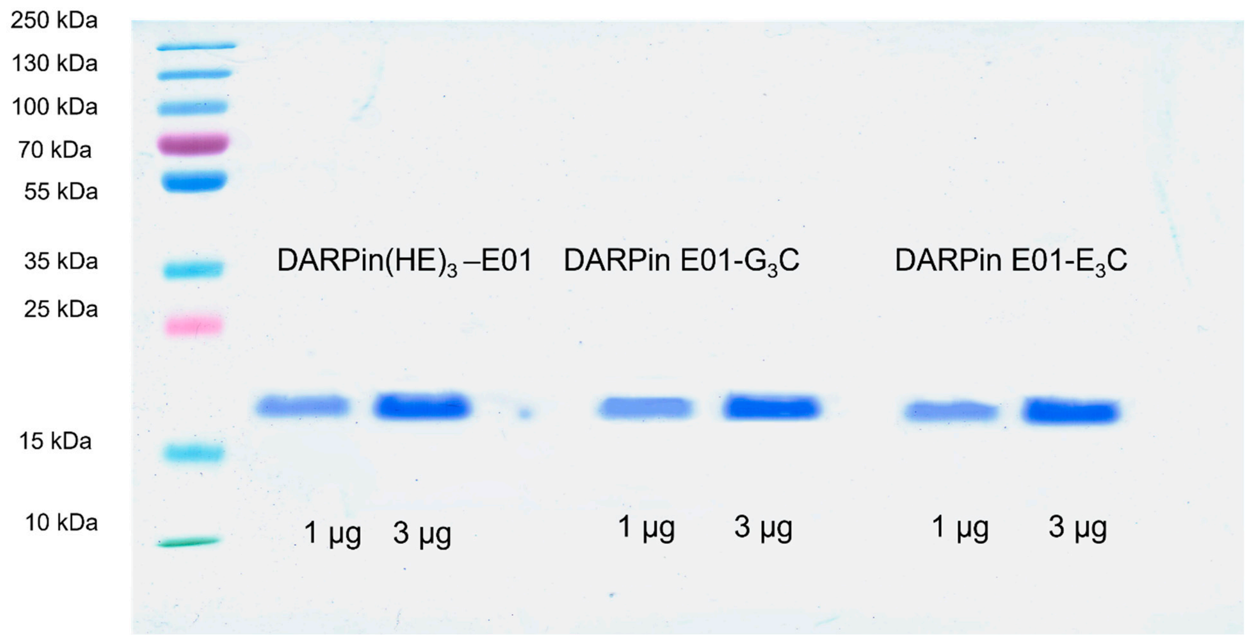

Figure S1. The purity of all DARPin E01 variants by SDS-PAGE.

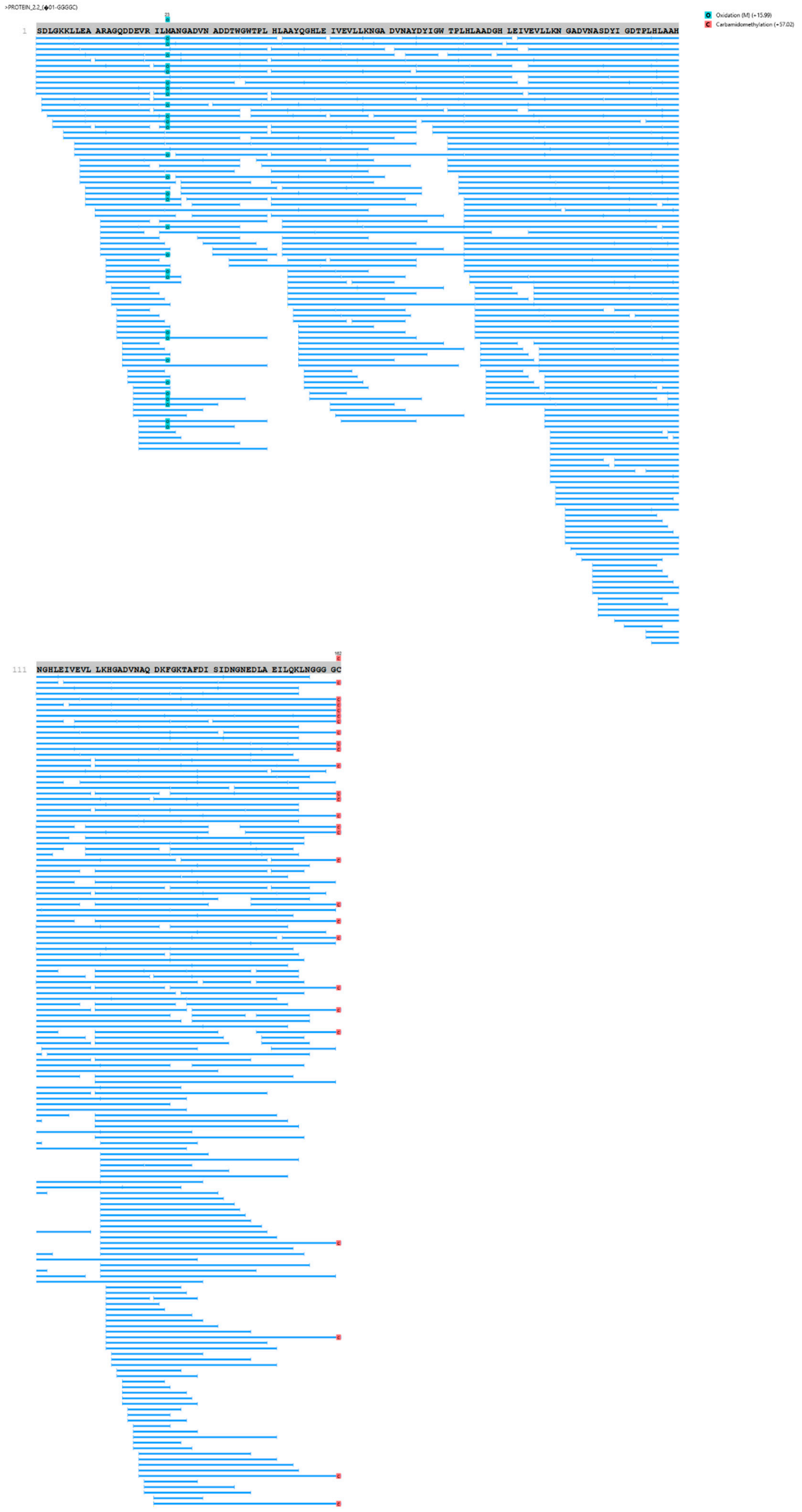

>PROTEIN_2.2_(�01-GGGGC)
Oxidation (M) (+15.99)
Carbamidomethylation (+57.02)
23
1 SDLGKKLLEA ARAGQDDEVR ILMANGADVN ADDTWGWTPL HLAAYQGHLE IVEVLLKNGA DVNAYDYIGW TPLHLAADGH LEIVEVLLKN GADVNASDYI GDTPLHLAAH
162
111 NGHLEIVEVL LKHGADVNAQ DKFGKTAFDI SIDNGNEDLA EILQKLNGGG GC

Figure S2. Overlap of the amino acid sequence of the E01-G$_3$C protein by identified peptide fragments. Amino acids are designated in accordance with the IUPAC nomenclature (alphabetic designations are given). Amino acids undergoing modification during the analysis are indicated by a red square ("C" - carbamidomethylation) or a blue square ("D" - deamidation)

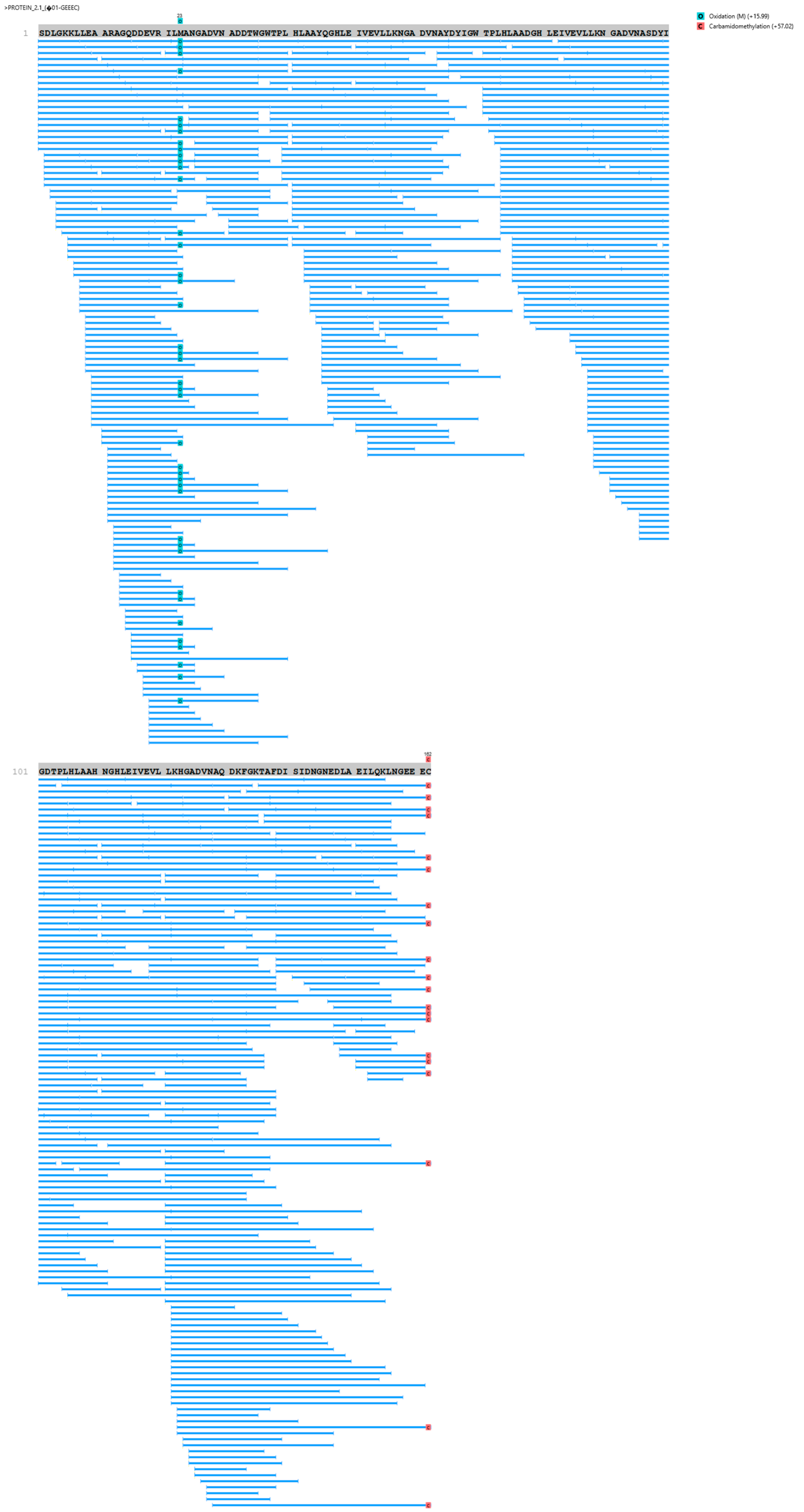
>PROTEIN_2.1_(�01-GEEEC)
Oxidation (M) (+15.99)
Carbamidomethylation (+57.02)
23
1 SDLGKKLLEA ARAGQDDEVR ILMANGADVN ADDTWGWTPL HLAAYQGHLE IVEVLLKNGA DVNAYDYIGW TPLHLAADGH LEIVEVLLKN GADVNASDYI
162
101 GDTPLHLAAH NGHLEIVEVL LKHGADVNAQ DKFGKTAFDI SIDNGNEDLA EILQKLNGEE EC

Figure S3. Overlap of the amino acid sequence of the E01-E3C protein by identified peptide fragments. Amino acids are designated in accordance with the IUPAC nomenclature (alphabetic designations are given). Amino acids undergoing modification during the analysis are indicated by a red square ("C" - carbamidomethylation) or a blue square ("O" - oxidation)

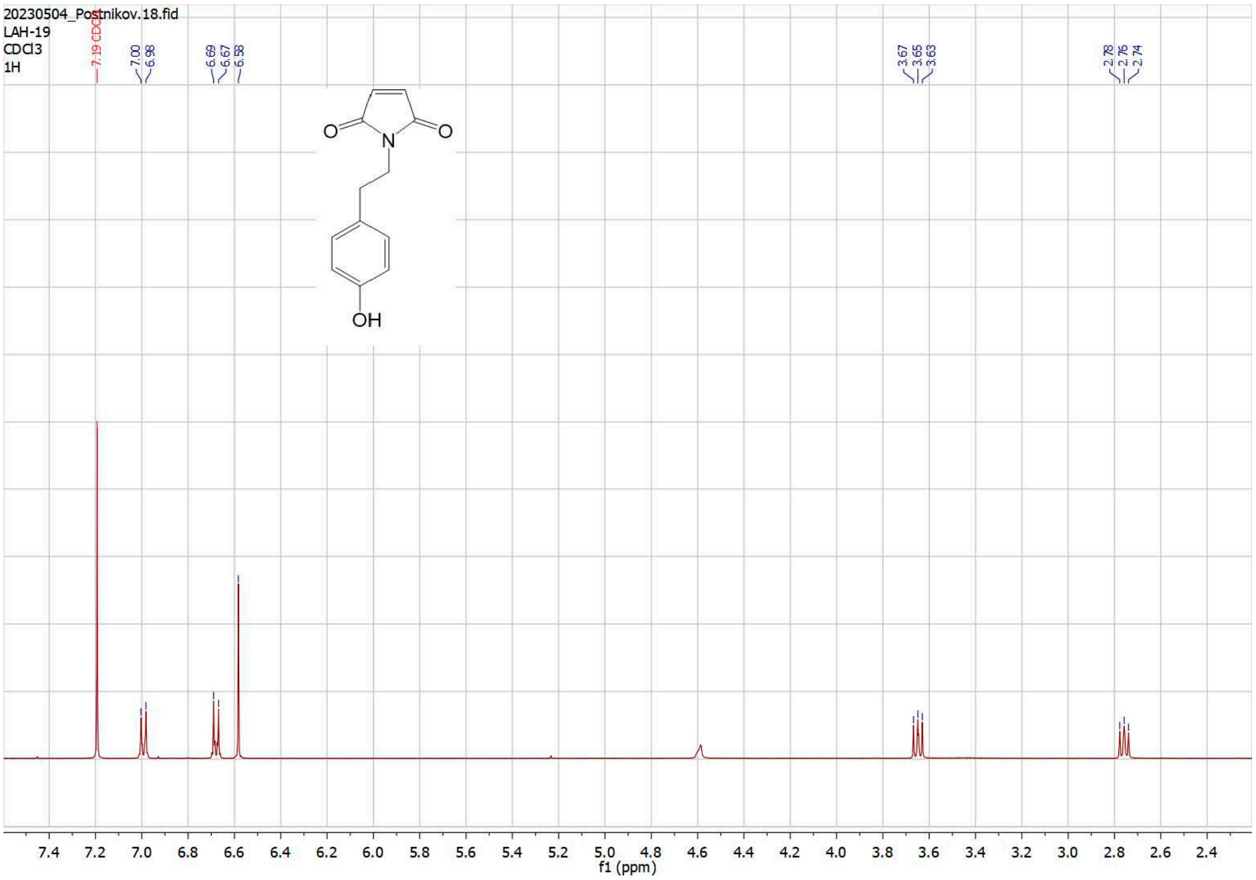

Figure S4. ^{1}H NMR spectra of ((4-hydroxyphenyl)ethyl)maleimide

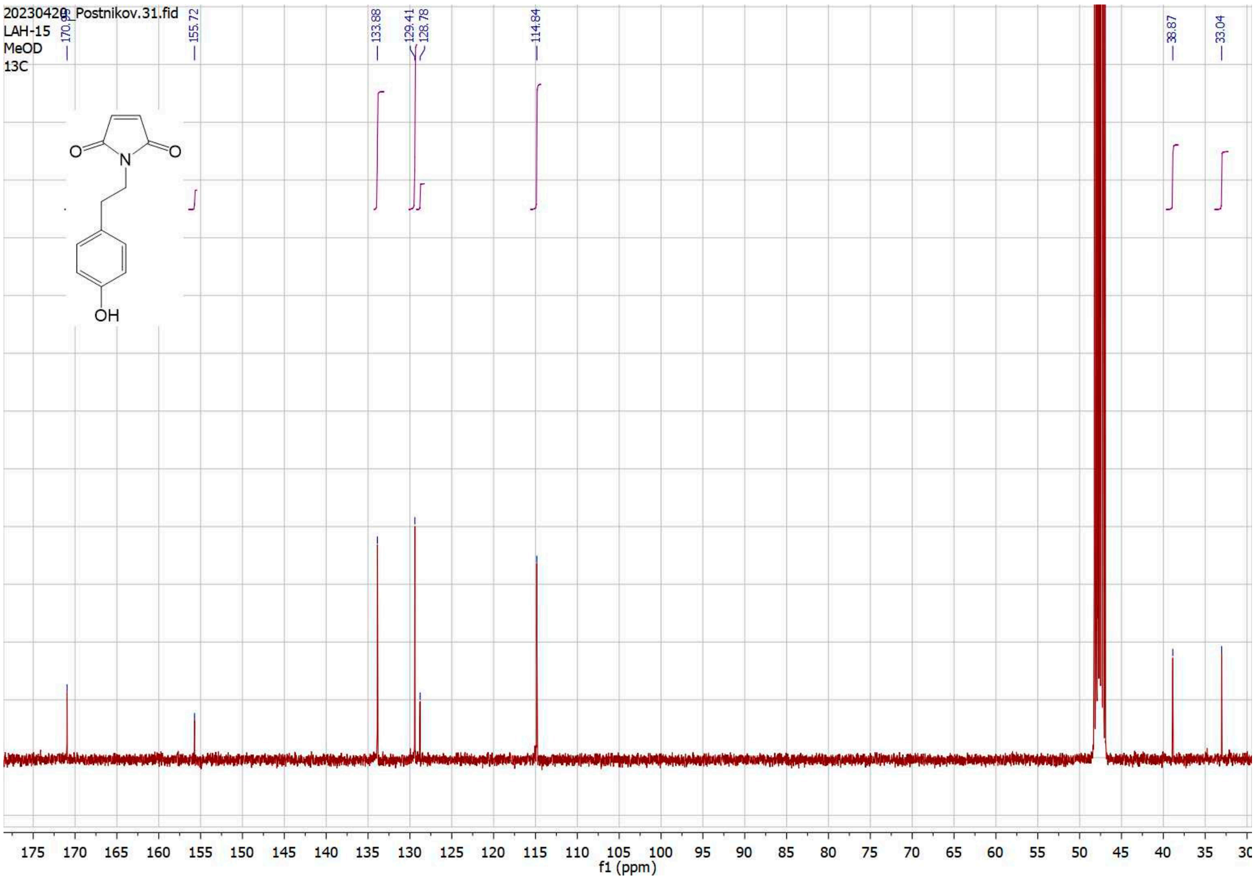

Figure S5. ^{13}C NMR spectra of ((4-hydroxyphenyl)ethyl)maleimide

Table S1. Comparative biodistribution [^{123}I]I-(HE)$_3$-E01-PIB, [^{123}I]I-E01-E$_3$C-HPEM and [^{123}I]I-E01-G$_3$C-HPEM variant radiolabelled with [^{123}I]I in Nu/j mice bearing HER1-expressing A-431 and Ramos xenografts. Data are presented as mean %ID/g ± SD for four mice. Data for the rest of the GI tract, contents, and the rest of the body are presented as %ID per whole sample.

	**[^{123}I]I-(HE)$_3$-E01-PIB**		**[^{123}I]I-E01-E$_3$C-HPEM**		**[^{123}I]I-E01-G$_3$C-HPEM**	
**Tissue**	A-431	Ramos	A-431	Ramos	A-431	Ramos
**Blood**	0.29 ± 0.03[b]	0.25 ± 0.02	0.21 ± 0.03	0.2 ± 0.2	0.2 ± 0.1	0.5 ± 0.1
**Salivary glands**	0.12 ± 0.02[a.b]	0.13 ± 0.08	0.6 ± 0.2	0.7 ± 0.3	0.6 ± 0.3	0.4 ± 0.1
**Lung**	0.18 ± 0.03	0.2 ± 0.1	0.3 ± 0.1	0.2 ± 0.1	0.2 ± 0.02	0.2 ± 0.1
**Liver**	0.41 ± 0.15[a]	0.6 ± 0.2	2.4 ± 1.1	2. ± 0.5	1.5 ± 0.3	1.7 ± 1.1
**Spleen**	0.17 ± 0.04[a]	0.2 ± 0.01	0.8 ± 0.2[c]	0.2 ± 0.1	0.33 ± 0.08	0.3 ± 0.1
**Pancreas**	0.08 ± 0.02[a]	0.09 ± 0.02	0.24 ± 0.05[c]	0.1 ± 0.01	0.08 ± 0.04	0.2 ± 0.1
**Small intestine**	0.2 ± 0.1[a.b]	0.2 ± 0.1	1.3 ± 0.4[c]	0.8 ± 0.3	0.8 ± 0.1	0.4 ± 0.2
**Stomach**	0.23 ± 0.05[a.b]	0.2 ± 0.1	2.3 ± 0.9	1.9 ± 1.6	1.7 ± 0.5	2.3 ± 0.4
**Kidney**	6.1 ± 1.2[a.b]	5 ± 1	1.1 ± 0.5	0.6 ± 0.2	0.8 ± 0.3	0.4 ± 0.1
**Tumour**	1.6 ± 0.1	0.14 ± 0.03	1.1 ± 0.3	0.09 ± 0.02	1.7 ± 0.6	0.04 ± 0.01
**Muscle**	0.06 ± 0.03	0.039 ± 0.003	0.08 ± 0.02	0.08 ± 0.02	0.05 ± 0.02	0.2 ± 0.1
**Bone**	0.35 ± 0.06	0.35 ± 0.11	0.5 ± 0.1[c]	0.4 ± 0.1	0.3 ± 0.1	0.5 ± 0.1
**GI tract with content**	2.7 ± 1.4[a.b]	2.4 ± 0.8	21.6 ± 2.8	17.0 ± 4.0	16.0 ± 4.0	17.0 ± 3.0
**Rest of the body**	1.4 ± 0.2[a.b]	1.25 ± 0.07	3.5 ± 0.3[c]	3.0 ± 1.0	2.6 ± 0.2	2.8 ± 0.5

[a]Significant difference ($p < 0.05$) between **[^{123}I]I-(HE)$_3$-E01-PIB** vs. **[^{123}I]I-E01-E$_3$C-HPEM** (unpaired t-test).

[b]Significant difference ($p < 0.05$) between **[^{123}I]I-(HE)$_3$-E01-PIB** vs. **[^{123}I]I-E01-G$_3$C-HPEM** (unpaired t-test).

[c]Significant difference ($p < 0.05$) between **[^{123}I]I-E01-E$_3$C-HPEM** vs. **[^{123}I]I-E01-G$_3$C-HPEM**

Table S2. Tumor-to-organ ratios [^{123}I]I-(HE)$_3$-E01-PIB, [^{123}I]I-E01-E$_3$C-HPEM and [^{123}I]I-E01-G$_3$C-HPEM in Nu/J mice bearing A-431 xenografts at 4 h pi. Data are presented as mean ± SD for four mice.

Tissue	[^{123}I]I-(HE)$_3$-E01-PIB	[^{123}I]I-E01-E$_3$C-HPEM	[^{123}I]I-E01-G$_3$C-HPEM
**Blood**	5.0 ± 0.4^{b}	5.1 ± 1.6^{c}	11.1 ± 4.1
**Salivary glands**	13.5 ± 1.7$^{a.b}$	2.1 ± 0.9	3.2 ± 1.5
**Lung**	8.7 ± 1.6$^{a.}$	4.3 ± 1.8	6.8 ± 2.1
**Liver**	4.2 ± 1.3$^{a.b}$	0.5 ± 0.1^{c}	1.2 ± 0.6
**Spleen**	9.3 ± 1.7$^{a.b}$	1.4 ± 0.5^{c}	5.2 ± 1.7
**Pancreas**	19.3 ± 4.0$^{a.b}$	4.9 ± 2.4^{c}	29.1 ± 0.6
**Small intestine**	8.4 ± 3.3$^{a.b}$	0.78 ± 0.04^{c}	2.1 ± 0.7
**Stomach**	7.2 ± 2.1$^{a.b}$	0.5 ± 0.2	1.03 ± 0.37
**Kidney**	0.3 ± 0.1^{b}	1.1 ± 0.4	2.4 ± 1.6
**Muscle**	33.0 ± 12.0^{a}	13.9 ± 1.5	37.0 ± 27.0
**Bone**	4.6 ± 0.9^{a}	2.1 ± 0.5^{c}	6.8 ± 3.2

^{a}Significant difference ($p < 0.05$) between **[^{123}I]I-(HE)$_3$-E01-PIB** vs. **[^{123}I]I-E01-E$_3$C-HPEM** (unpaired t-test).

^{b}Significant difference ($p < 0.05$) between **[^{123}I]I-(HE)$_3$-E01-PIB** vs. **[^{123}I]I-E01-G$_3$C-HPEM** (unpaired t-test).

^{c}Significant difference ($p < 0.05$) between **[^{123}I]I-E01-E$_3$C-HPEM** vs. **[^{123}I]I-E01-G$_3$C-HPEM**
